# Supplementary figures and images for: Antimicrobial Functions of Lactoferrin Promote Genetic Conflicts in Ancient Primates and Modern Humans
Source: PLoS Genet. 2016 May 20;12(5):e1006063. doi: 10.1371/journal.pgen.1006063 (PMC4874600; doi:10.1371/journal.pgen.1006063)

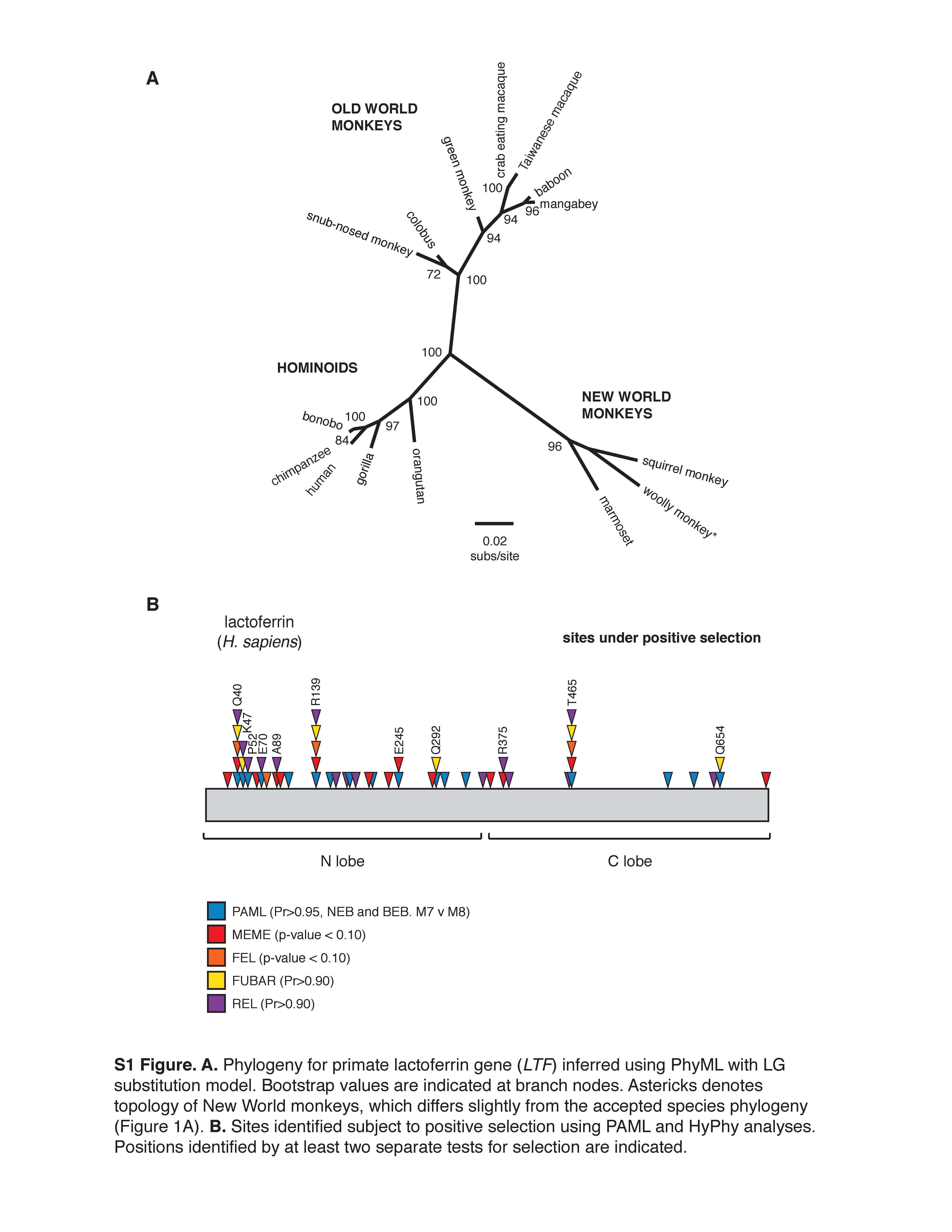

Supplement: S1 Fig — (TIFF) [file pgen.1006063.s001.tiff]

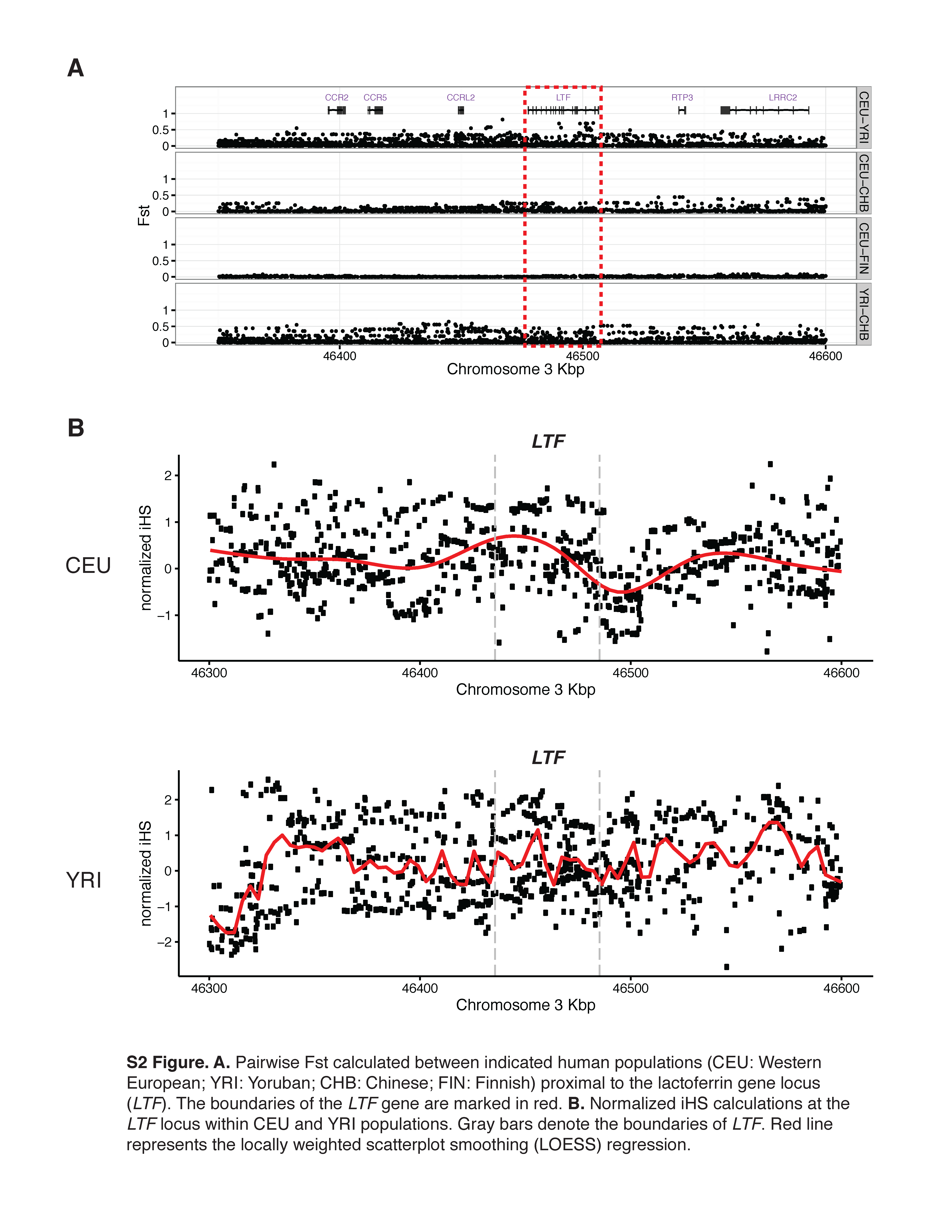

Supplement: S2 Fig — (TIFF) [file pgen.1006063.s002.tiff]

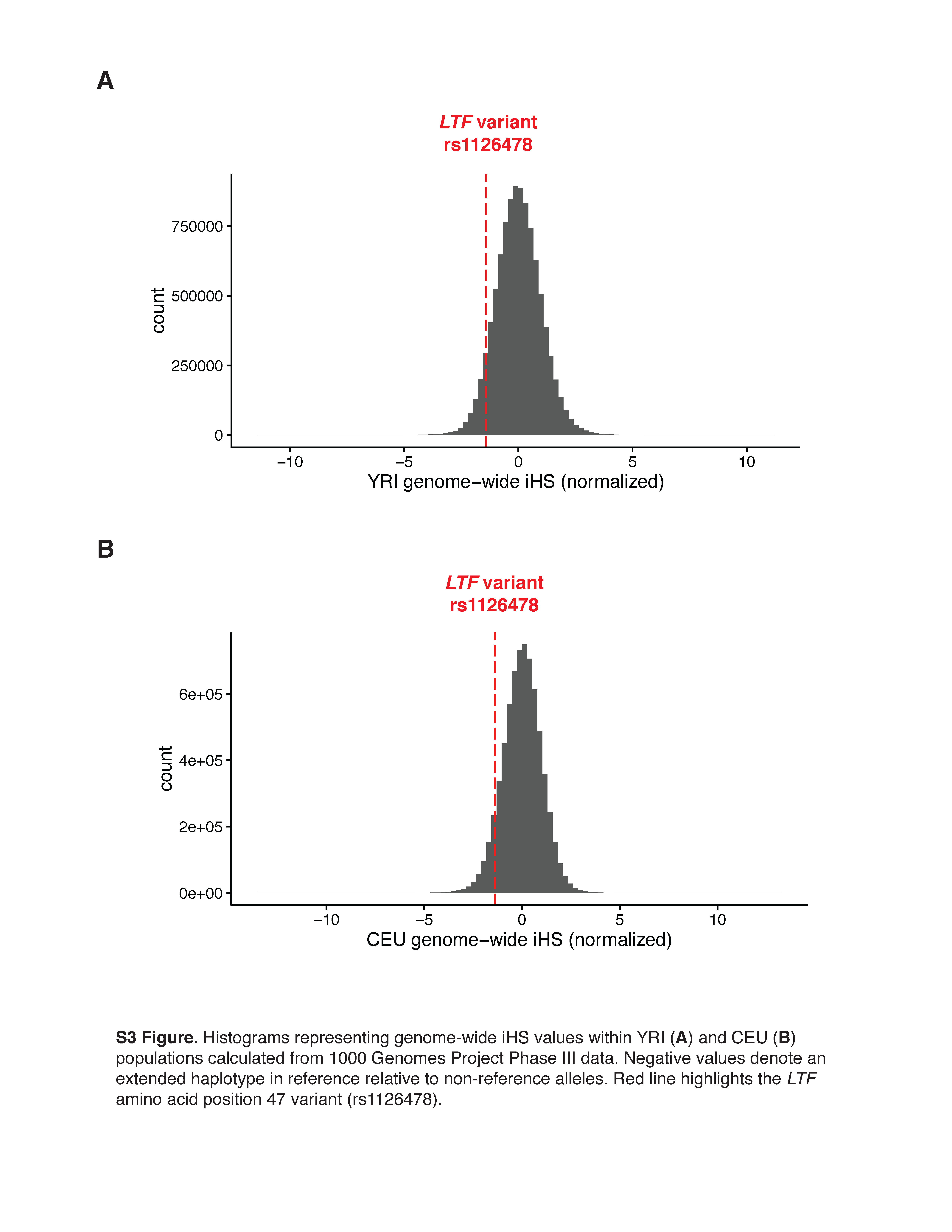

Supplement: S3 Fig — (TIFF) [file pgen.1006063.s003.tiff]

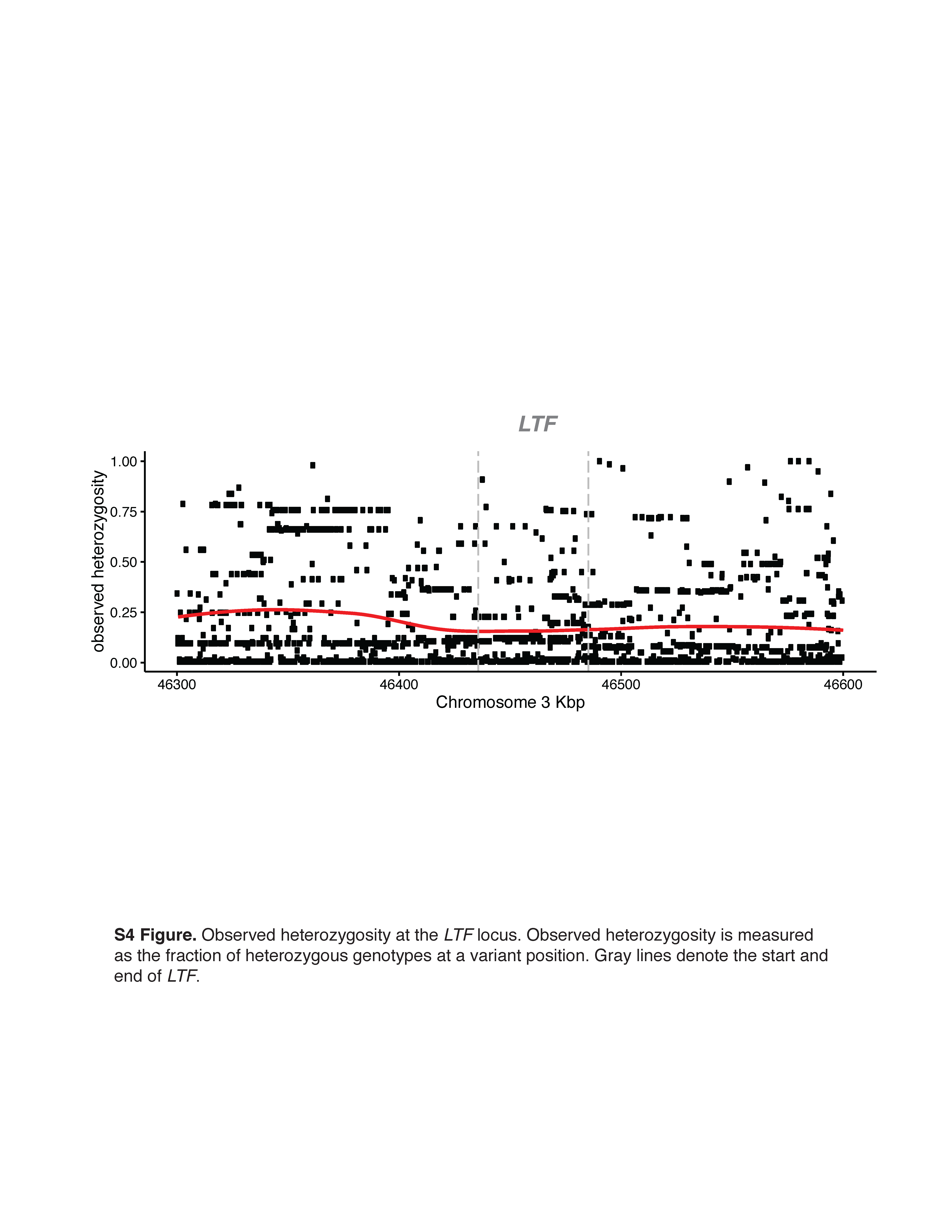

Supplement: S4 Fig — (TIFF) [file pgen.1006063.s004.tiff]

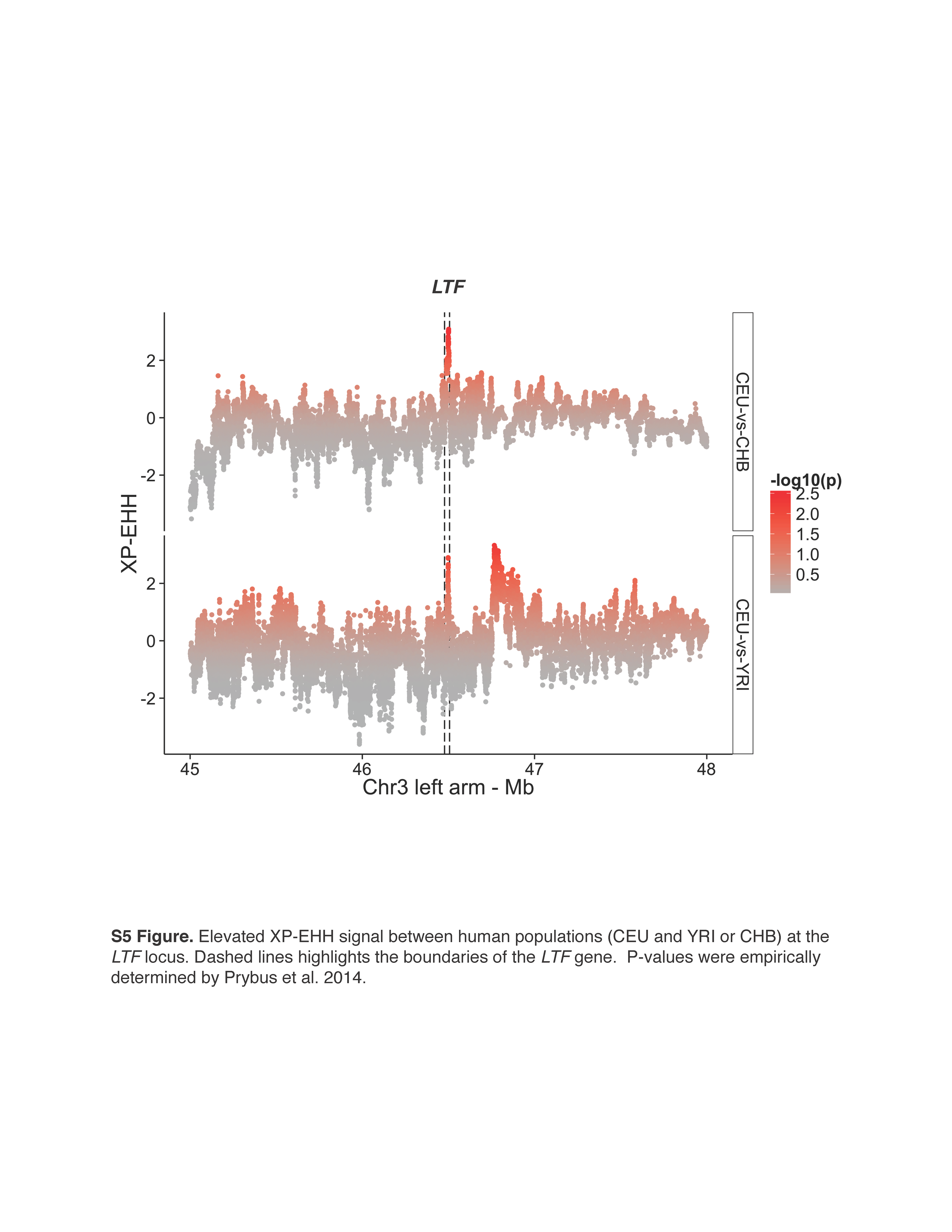

Supplement: S5 Fig — (TIFF) [file pgen.1006063.s005.tiff]

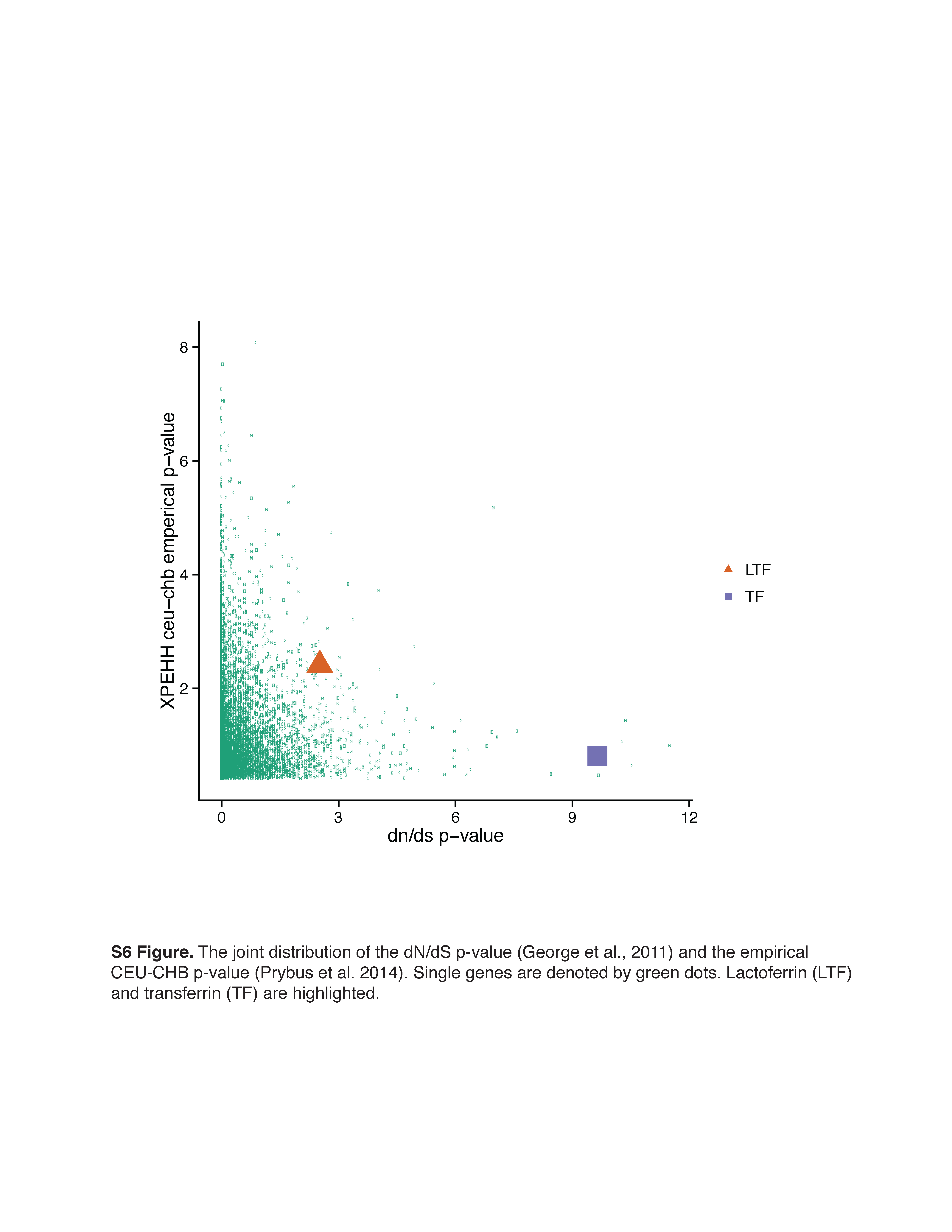

Supplement: S6 Fig — (TIFF) [file pgen.1006063.s006.tiff]

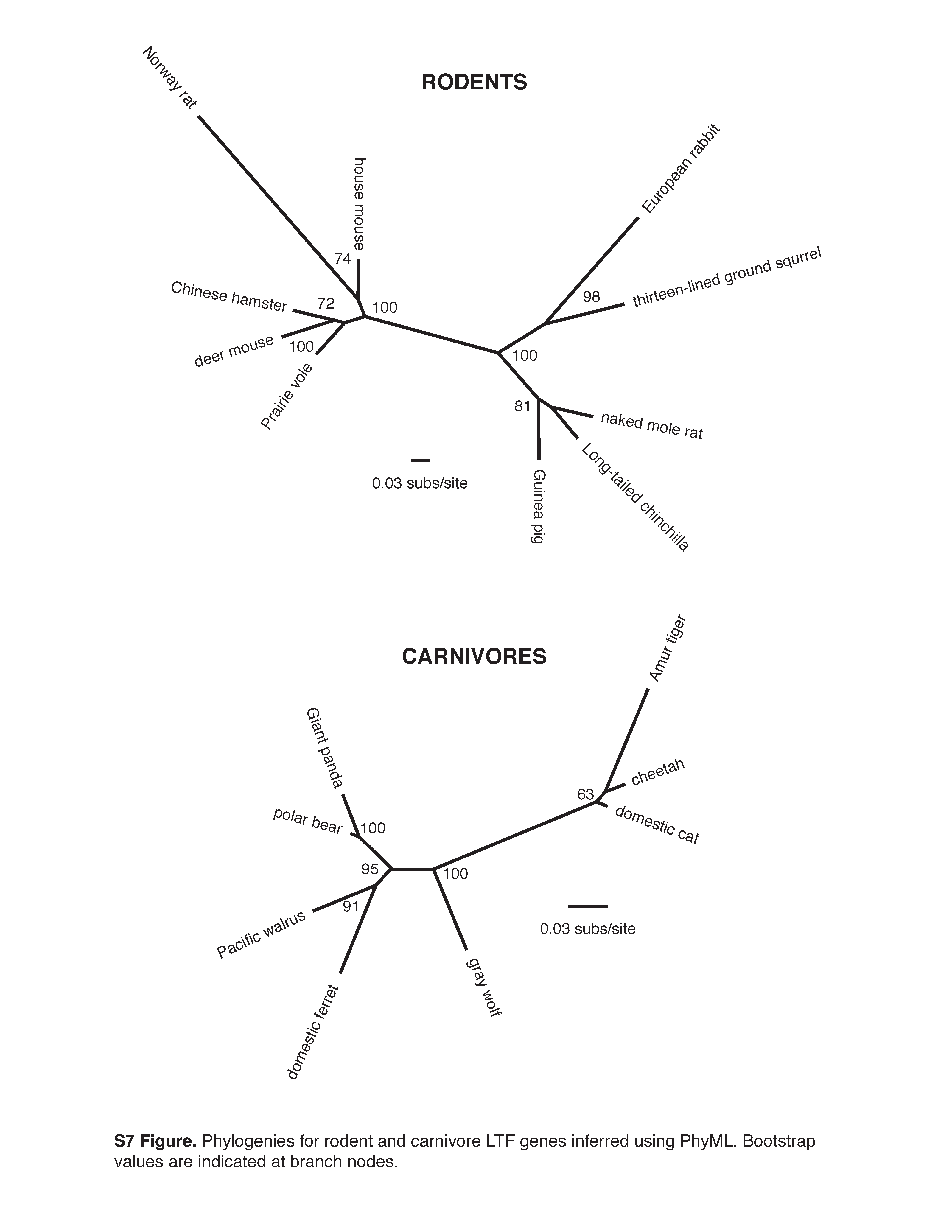

Supplement: S7 Fig — (TIFF) [file pgen.1006063.s007.tiff]

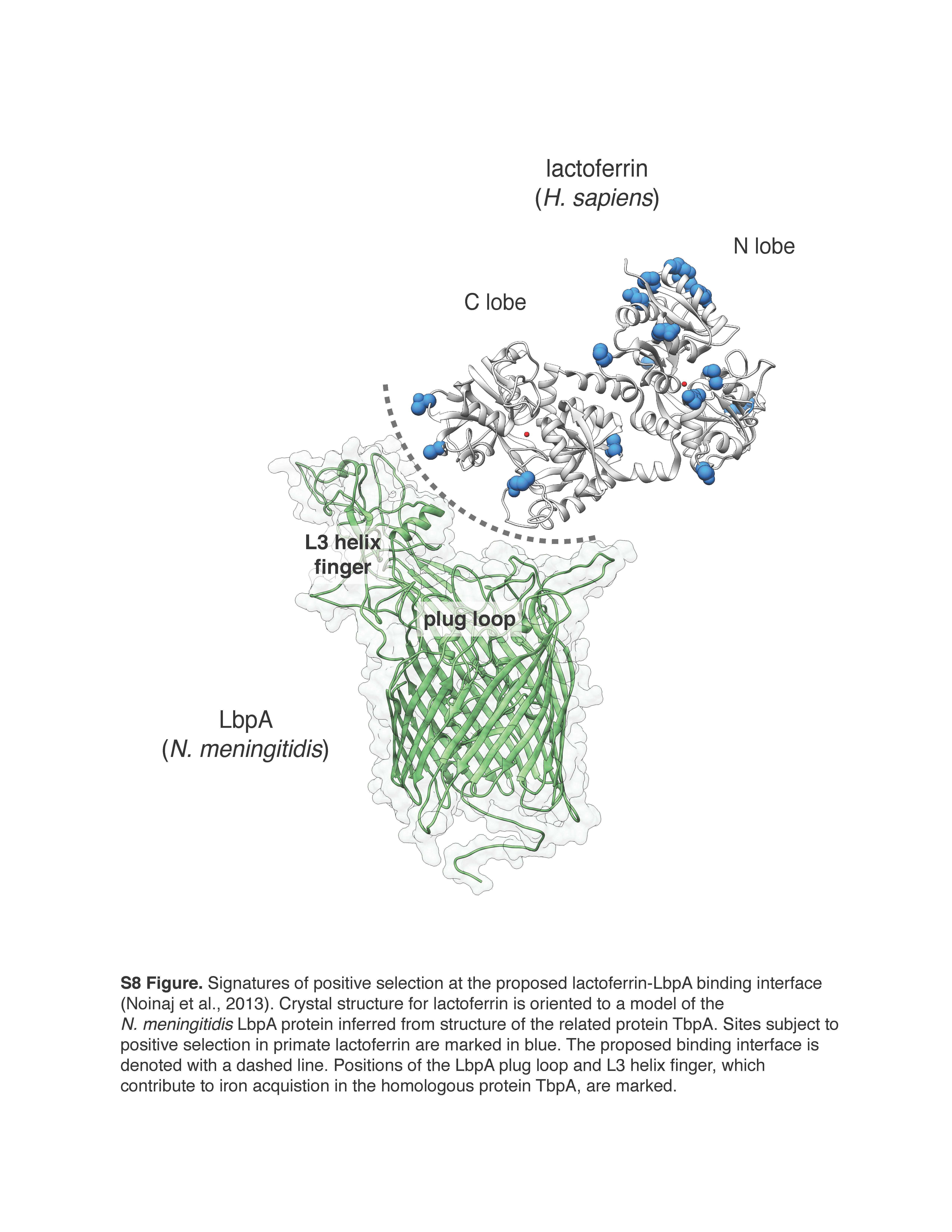

Supplement: S8 Fig — (TIFF) [file pgen.1006063.s008.tiff]
