# Supplementary material for: Antimicrobial Functions of Lactoferrin Promote Genetic Conflicts in Ancient Primates and Modern Humans
Source: PLoS Genet. 2016 May 20;12(5):e1006063. doi: 10.1371/journal.pgen.1006063 (PMC4874600; doi:10.1371/journal.pgen.1006063)
Supplement: S1 Table — (DOCX) [file pgen.1006063.s009.docx]

**S1 Table.** Likelihood ratio test statistics for models of variable selection along branches of the primate lactoferrin phylogeny using PAML.

|  | **lnL*** | **2δ** | **Df**** | **p-value** |
| --- | --- | --- | --- | --- |
| Model 0  Same dN/dS for all branches | -6357.95 |  |  |  |
| Model 1  Different dN/dS ratio for different branches | -6267.33 | 181.24 | 26 | <0.0001 |

* likelihood score

** degrees of freedom, equal to one less than the total number of branches in the phylogeny.
