# Supplementary material for: Antimicrobial Functions of Lactoferrin Promote Genetic Conflicts in Ancient Primates and Modern Humans
Source: PLoS Genet. 2016 May 20;12(5):e1006063. doi: 10.1371/journal.pgen.1006063 (PMC4874600; doi:10.1371/journal.pgen.1006063)
Supplement: S2 Table — (DOCX) [file pgen.1006063.s010.docx]

**S2 Table.** Summary of positive selection in primate lactoferrin using PAML. Analyses were performed using two independent codon models (F3X4, F61) which describe the frequency and rate of substitutions. Selection was inferred by comparing likelihood scores between models that allow for selection (M2, M8) relative models which exclude selection (M1, M7) in this gene.

|  | **Codon freq.** | ***M1-M2*** | | ***M7-M8*** | | **Tree length** | **dN/dS (%)** |
| --- | --- | --- | --- | --- | --- | --- | --- |
|  |  | **2δ** | **p-value** | **2δ** | **p-value** |  |  |
| **Whole gene** | F3X4 | 16.9 | 0.00021 | 18.6 | <0.0001 | 0.90 | 1.8 (22.5) |
|  | F61 | 21.5 | <0.0001 | 23.0 | <0.0001 | 0.89 | 1.9 (22.2) |
| **N-lobe** | F3X4 | 14.3 | 0.00079 | 14.8 | 0.00060 | 0.99 | 2.1 (20.6) |
|  | F61 | 17.8 | 0.00014 | 18.1 | 0.00012 | 0.96 | 2.0 (26.7) |
| **C-lobe** | F3X4 | 4.2 | 0.12 | 4.3 | 0.12 | 0.81 | 1.6 (20.3) |
|  | F61 | 6.5 | 0.038 | 7.3 | 0.025 | 0.80 | 1.9 (17.2) |
