# Supplementary material for: Antimicrobial Functions of Lactoferrin Promote Genetic Conflicts in Ancient Primates and Modern Humans
Source: PLoS Genet. 2016 May 20;12(5):e1006063. doi: 10.1371/journal.pgen.1006063 (PMC4874600; doi:10.1371/journal.pgen.1006063)
Supplement: S4 Table — (DOCX) [file pgen.1006063.s012.docx]

**S4 Table.** Lactoferrin whole gene log likelihood scores and parameter estimates for four models of variable ω among sites assuming the F3X4 model of codon frequencies (PAML). Amino acid positions shown are for human lactoferrin.

| **Site model** | **Parameter estimates** | **Sites* with ω**  ** | **lnL** |
| --- | --- | --- | --- |
| **M1: neutral** | (ω0=0) *f*0= 0.635   (ω1=1) *f*1=  0.365  branch ω (mean)= 0.365 |  | -6267.33 |
| **M2: selection** | (ω 0=0.06) *f*0= 0.731  (ω 1=1) *f*1= 0.122  **(ω 2=2.1) *f*2=0.146**  branch ω (mean)= 0.466 | M623 | -6258.87 |
| **M7: β** | *p*= 0.005  *q*=  0.0074  branch ω (mean)= 0.40 |  | -6268.21 |
| **M8: β and ω** | *p*= 8.415  *q*= 99.00 (*f*0= 0.775)  **ω 1= 1.8 ( *f*1= 0.225)**  branch ω (mean)= 0.465 | Q40  K47  P52  E70  R105  R139  T158  G183  S210  E245  Q292  K304  P331  T465  A588  M622  Q654 | -6258.92 |

* posterior probabilities >0.95 by both Naïve Empirical Bayes (NEB) and Bayes Empirical Bayes analysis.

** ω = dN/dS
