# Supplementary material for: Antimicrobial Functions of Lactoferrin Promote Genetic Conflicts in Ancient Primates and Modern Humans
Source: PLoS Genet. 2016 May 20;12(5):e1006063. doi: 10.1371/journal.pgen.1006063 (PMC4874600; doi:10.1371/journal.pgen.1006063)
Supplement: S5 Table — (DOCX) [file pgen.1006063.s013.docx]

| **Model** | **Sites with evidence of positive selection (p-value)** |
| --- | --- |
| MEME | Q26 (0.018)  Q40 (0.089)  Q63 (0.087)  A89 (0.097)  K92 (0.020)  R139 (0.00020)  E206 (0.0030)  E230 (0.084)  E245 (0.0087)  N288 (0.019)  R361 (0.050)  R375 (0.015)  T465 (0.035)  R709 (0.014) |
| FEL | Q40 (0.075)  V76 (0.097)  R139 (0.044)  T465 (0.034) |

**S5 Table.** Summary of positive selection in primate lactoferrin (MEME, FEL). Amino acid positions shown are for human lactoferrin.
