# Supplementary material for: Antimicrobial Functions of Lactoferrin Promote Genetic Conflicts in Ancient Primates and Modern Humans
Source: PLoS Genet. 2016 May 20;12(5):e1006063. doi: 10.1371/journal.pgen.1006063 (PMC4874600; doi:10.1371/journal.pgen.1006063)
Supplement: S6 Table — (DOCX) [file pgen.1006063.s014.docx]

**S6 Table.** Summary of positive selection in primate lactoferrin using FUBAR and REL algorithms. Amino acid positions shown are for human lactoferrin.

| **Model** | **Sites with evidence of diversifying selection** | **Posterior probability** | **Bayes Factor** |
| --- | --- | --- | --- |
| FUBAR | Q40  K47  R139  Q292  T465  Q654 | 0.90  0.91  0.96  0.93  0.97  0.92 | 17.1  18.3  49.2  23.6  64.6  22.5 |
| REL | Q40  K47  P52  E70  A89  R139  P163  K182  G183  A193  K352  R375  G382  R462  T465  D648 | 0.94  0.93  0.94  0.95  0.93  0.94  0.94  0.93  0.95  0.92  0.92  0.93  0.93  0.92  0.95  0.93 | 67.6  57.7  72.1  83.0  61.4  71.2  67.9  57.6  77.3  51.9  52.1  60.0  58.6  52.2  77.4  61.6 |
