# Supplementary material for: Antimicrobial Functions of Lactoferrin Promote Genetic Conflicts in Ancient Primates and Modern Humans
Source: PLoS Genet. 2016 May 20;12(5):e1006063. doi: 10.1371/journal.pgen.1006063 (PMC4874600; doi:10.1371/journal.pgen.1006063)
Supplement: S7 Table — (DOCX) [file pgen.1006063.s015.docx]

**Supplementary Table 7.** BUSTED likelihood ratio test statistics for gene-wide episodic diversifying selection in primate lactoferrin.

| **Model** | **log L** | **AICc** | **Tree length** | **LRT p-value** |
| --- | --- | --- | --- | --- |
| **Unconstrained** | -6305.60 | 12703.61 | 0.91 | 0.001 |
| **Constrained** | -6312.57 | 12715.52 | 0.86 |  |
