# Supplementary material for: Antimicrobial Functions of Lactoferrin Promote Genetic Conflicts in Ancient Primates and Modern Humans
Source: PLoS Genet. 2016 May 20;12(5):e1006063. doi: 10.1371/journal.pgen.1006063 (PMC4874600; doi:10.1371/journal.pgen.1006063)
Supplement: S8 Table — (DOCX) [file pgen.1006063.s016.docx]

| **Designation** | **MAF** | **mRNA** | | **protein** | |
| --- | --- | --- | --- | --- | --- |
|  |  | **Position** | **Allele change** | **Positon (lobe)** | **Residue change** |
| rs1126477 | 0.47 | 360 | GCC 🡺 ACC | 29 (N) | A [Ala] 🡺 T [Thr] |
| rs1126478 | 0.37 | 415 | AGA 🡺 AAA | 47 (N) | R [Arg] 🡺 K [Lys] |
| rs141854619 | 0.01 | 460 | ATC 🡺 AAC | 62 (N) | I [Ile] 🡺 N [Asn] |
| rs60938611 | 0.06 | 853 | GCG 🡺 GTG | 193 (N) | A [Ala] 🡺 V [Val] |
| rs2073495 | 0.28 | 2012 | GAG 🡺 GAC | 579 (C) | E [Glu] 🡺 D [Asp] |
| rs9110 | 0.48 | 2169 | TTG 🡺 ATG | 632 (C) | L [Leu] 🡺 M [Met] |

**S8 Table.** Summary of abundant (>1% MAF) lactoferrin missense variants in the human population. Allele frequencies are reported from 1000 Genomes project data (phase 3). MAF: minor allele frequency.
