# Supplementary material for: Antimicrobial Functions of Lactoferrin Promote Genetic Conflicts in Ancient Primates and Modern Humans
Source: PLoS Genet. 2016 May 20;12(5):e1006063. doi: 10.1371/journal.pgen.1006063 (PMC4874600; doi:10.1371/journal.pgen.1006063)
Supplement: S9 Table — (DOCX) [file pgen.1006063.s017.docx]

**S9 Table.** Allele frequencies for lactoferrin rs1126478 variant in 1000 Genomes database (phase 3).

| **Population** | **Allele frequency (K48)** | **Allele frequency (R48)** | **Observed heterozygosity** | **Expected heterozygosity** |
| --- | --- | --- | --- | --- |
| African (AFR) | 0.035874 | 0.964126 | 0.0691749 | 0.0687593 |
| East Asian (EAS) | 0.348544 | 0.651456 | 0.454122 | 0.425243 |
| South Asian (SAS) | 0.456186 | 0.543814 | 0.496161 | 0.453608 |
| European (EUR) | 0.651485 | 0.348515 | 0.454104 | 0.451485 |
| American (AMR) | 0.512784 | 0.487216 | 0.499673 | 0.451705 |
