# Supplementary material for: Antimicrobial Functions of Lactoferrin Promote Genetic Conflicts in Ancient Primates and Modern Humans
Source: PLoS Genet. 2016 May 20;12(5):e1006063. doi: 10.1371/journal.pgen.1006063 (PMC4874600; doi:10.1371/journal.pgen.1006063)
Supplement: S10 Table — (DOCX) [file pgen.1006063.s018.docx]

**S10 Table.** Summary of positive selection in rodent and carnivore lactoferrin using PAML. Analyses were performed using two independent codon models (F3X4, F61). Selection was inferred by comparing likelihood scores between models that allow for selection (M2, M8) relative models which exclude selection (M1, M7) in this gene. Tree length and dN/dS values are shown for the M8 calculations.

|  | **Codon freq.** | ***M1-M2*** | | ***M7-M8*** | | **Tree length** | **dN/dS (%)** |
| --- | --- | --- | --- | --- | --- | --- | --- |
|  |  | **2δ** | **p-value** | **2δ** | **p-value** |  |  |
| **Rodents (10 species)** | F3X4 | 9.0 | 0.011 | 31.0 | <0.0001 | 4.1 | 1.9 (5.1) |
|  | F61 | 0 | 1.0 | 24.3 | <0.0001 | 3.9 | 2.1 (4.1) |
| **Carnivores (8 species)** | F3X4 | 6.6 | 0.037 | 9.7 | 0.0079 | 1.1 | 4.3 (1.7) |
|  | F61 | 4.5 | 0.10 | 5.7 | 0.059 | 1.1 | 4.5 (1.2) |
