# Supplementary material for: Antimicrobial Functions of Lactoferrin Promote Genetic Conflicts in Ancient Primates and Modern Humans
Source: PLoS Genet. 2016 May 20;12(5):e1006063. doi: 10.1371/journal.pgen.1006063 (PMC4874600; doi:10.1371/journal.pgen.1006063)
Supplement: S1 Data — (DOCX) [file pgen.1006063.s019.docx]

Lactoferrin cDNA: CLUSTAL O(1.2.1) multiple sequence alignment

human ATGAAACTTGTCTTCCTCGTCCTGCTGTTCCTCGGGGCCCTCGGACTGTGTCTGGCTGGC 60

chimpanzee ATGAAACTTGTCTTCCTCGTCCTGCTCTTCCTCGGGGCCCTCGGACTGTGTCTGGCTGGC 60

bonobo ATGAAACTTGTCTTCCTCGTCCTGCTCTTCCTCGGGGCCCTCGGACTGTGTCTGGCTGGC 60

gorilla ATGAAACTTGTCTTCCCCGTCCTGCTGTTCCTCGGGGCCCTCGGACTGTGTCTAGCTGCC 60

orangutan ATGAAGCTTGTCTTCTCCGCCCTGCTGTTCCTCGGGGCCCTCGGACTGTGTCTGGCTGCC 60

CEmacaque ATGAAGCTTGTCTTCCTCGCCCTACTGTTCCTCGGGACCCTCGGACTGTGTCTGGCTGCC 60

Tmacaque ATGAAGCTTGTCTTCCTCGCCCTACTGTTCCTCGGGACCCTCGGACTGTGTCTGGCTGCC 60

baboon ATGAAGCTTGTCTTTCTCGCCCTACTGTTCCTCGGGACCCTCGGACTGTGTCTGGCTGCC 60

mangabey ATGAAGCTTGTCTTTCTCGCCCTACTGTTCCTCGGGACCCTCGGACTGTGTCTGGCTGCC 60

greenM ATGAAGCTTGTCTTCCTTGCCCTACTGTTCCTCGGGACCCTCGGACTGTGTCTGGCTGCC 60

colobus ATGAAGCTTGCCTTCCTCGCCCTGCTGTTCCTCGGGACCCTCGGACTGTGTCTGGCTGCC 60

SNmonkey ATGAAGCTTGTCTTCCTCGCCCTGCTGTTCCTCGGGACCCTCGGACTGTGTCTGGCTGCC 60

marmoset ATGAAGCTTGTCTTCCCCACCCTGCTGTTCCTCGGGGCCCTCGGACTGTGTCTGGCTGCC 60

squirrelM ATGAAGCTTGTCTTCCCCGCCCTGCTGTTCCTCGGGGCCCTCGGACTGTGTCTGGCTGCC 60

woollyM ATGAAGCTTGTCTTCCCTGCCCTACTGTTCCTCGGGGCCCTCGGACTGTGTCTGGCTGCC 60

*****.**** *** . ***.** *********.****************.**** *

human CGTAGGAGAA---GGAGTGTTCAGTGGTGCGCCGTATCCCAACCCGAGGCCACAAAATGC 117

chimpanzee CCTAGGAGAA---GGAGTGTTCAGTGGTGCACTGTATCCCAACCCGAGGCCACAAAATGC 117

bonobo CCTAGGAGAA---GGAGTGTTCAGTGGTGCACTGTATCCCAACCCGAGGCCACAAAATGC 117

gorilla CCTAGGAGAA---GGAGTGTTCGATGGTGCACCGTATCCCAACCTGAGGCCACAAAATGC 117

orangutan CCTAGGAGAAGGAGGAGTGTTCGATGGTGCACCGTATCCCAACCCGAGGCCACAAAATGC 120

CEmacaque CGTAGG---A---GGAGTGTTCGATGGTGCGCTGTATCCAAACCCGAGGCCACAAAATGC 114

Tmacaque CGTAG------GAGGAGTGTTCGATGGTGCGCTGTATCCAAACCCGAGGCCACAAAATGC 114

baboon CGTAG------GAGGAGTGTTCGATGGTGCGCTGTATCCAAACCCGAGGCCACAAAATGC 114

mangabey CGTAGG---A---GGAGTGTTCGATGGTGCGCTGTATCCAAACCCGAGGCCACAAAATGC 114

greenM CGTAG------GAGGAGTGTTCGATGGTGCACTGTATCCCAACCCGAGGCCACAAAATGC 114

colobus CGTAGG---A---GGAGTGTTCGATGGTGCACTGTATCCCAACCCGAGGCCACAAAATGC 114

SNmonkey CGTAGGA------GGAGTGTTCGATGGTGCACTGTATCCCAACCCGAGGCCACAAAATGC 114

marmoset CCTAGG---G---GGAGCGTTCGATGGTGCACCGTATCCAAACCCGAGGCCACAAAATGC 114

squirrelM CCTAGG---G---GGAGCGTTCGATGGTGCACCGTATCCAAACCTGAGGCCACAAAATGC 114

woollyM CCTAGG---G---GGAGCGTTCGATGGTGCACCGTATCCAAACCCGAGGCCACAAAATGC 114

* *** **** ****..******.* ******.**** ***************

human TTCCAATGGCAAAGGAATATGAGAAAAGTGCGTGGCCCTCCTGTCAGCTGCATAAAGAGA 177

chimpanzee TTCCGATGGCAAAGGAATATGAGAAGAGTGCGTGGCCCTCCTGTCAGCTGCATAAAGAGA 177

bonobo TTCCGATGGCAAAGGAATATGAGAAGAGTGCGTGGCCCTCCTGTCAGCTGCATAAAGAGA 177

gorilla TTCCGATGGCAAAGGAATATGAAAAGAGTGCGTGGCCCTCCTGTCAGCTGCATAAAGAGA 177

orangutan TTCCGATGGCAAAGGAATATGAGAAGAGTGCGTGGCCCTCCTGTCAGCTGCATAAAGACA 180

CEmacaque TCCCAATGGCAAAGGAATCTGAGAAGAGTGCGTGGCCCTCCTGTCAGCTGCATAAAGAGA 174

Tmacaque TCCCAATGGCAAAGGAATCTGAGAAGAGTGCGTGGCCCTCCTGTCAGCTGCATAAAGAGA 174

baboon TCCCAATGGCAAAGGAATCTGAGAAGAGTGCGTGGCCCTCCTGTCAGCTGCATAAAGAGA 174

mangabey TCCCAATGGCAAAGGAATCTGAGAAGTGTGCGTGGCCCTCCTGTCAGCTGCATAAAGAGA 174

greenM TCCCAATGGCAAGGGAATCTGAGAAGAGTGCGTGGCCCTCCTGTCAGCTGCATAAAGAGA 174

colobus TCCCAATGGCAAAGGAATCTGAGAAGAGTGCTTGGCCCTCCTGTCAGCTGCATAAAGAGA 174

SNmonkey TCCCAATGGCAAAGGAATCTGAGAAGAGTGCGTGGCCCTCCTGTCAGCTGCATAAAGAGA 174

marmoset ATCCAATGGCAAAGGAACCTGAGAAAAGTGGGTGGCCCTTCTGTCAGCTGCATAAAGAGA 174

squirrelM ATCCAATGGCAAAGGAACCTGAGAAAAGTGGGTGGTCCTCCTGTCAGCTGCATAAAGAGA 174

woollyM ATCAAATGGCAAAGGAACCTGAGAAATGTGGGTGGCCCTTTTGTCAGCTGCATAAAGAGA 174

: *..*******.**** .***.**.:*** *** *** ***************** *

human GACTCCCCCATCCAGTGTATCCAGGCCATTGCGGAAAACAGGGCCGATGCTGTGACCCTT 237

chimpanzee GACTCCCCCATCCAGTGTATCCAGGCCATTGCGGAAAACAGGGCCGATGCTGTGACCCTT 237

bonobo GACTCCCCCATCCAGTGTATCCAGGCCATTGCGGAAAACAGGGCCGATGCTGTGACCCTT 237

gorilla GACTCCCCCATCCAGTGTATCCAGGCCATTGCGGAAAACAGGGCCGATGCTGTGACCCTT 237

orangutan GACTCCCCCACCCAGTGTATCCAGGCCATTGCGGCAAACAGGGCCGATGCTGTGACCCTT 240

CEmacaque GCCTCCCCCACCAACTGTATCCAGGCCATTGCGGCAAACAGGGCCGATGCTATGACCCTT 234

Tmacaque GCCTCCCCCACCAACTGTATCCAGGCCATTGCGGCAAACAGGGCCGATGCTATGACCCTT 234

baboon GCCTCCCCCACCAACTGTATCCGGGCCATTGCGGCAAATAGGGCCGATGCTATGACCCTT 234

mangabey GCCTCCCCCACCAACTGTATCCGGGCCATTGCGGCAAATAGGGCCGATGCTATGACCCTT 234

greenM GCCTCCCCCACCAACTGTATCCAGGCCATTGCGGCAAACAAGGCCGATGCTATGACCCTT 234

colobus GCCTCCCCCACCAACTGTATCCAGGCCATTGCGACAAACAAGGCCGATGCTGTGACCCTT 234

SNmonkey GCCTCCCCCACCAAGTGTATCCAGGCCATTGCGACAAACGAGGCCGATGCTGTGACCCTT 234

marmoset GCCTCCCCAACCCAGTGTGTGGAAGCCATTGCGACAAACAAGGCTGATGCTGTGACCCTT 234

squirrelM ACCTCCCCAACCCAGTGTATGGAAGCCATTGCGACAAACAAGGCTGATGCTGTGACCCTT 234

woollyM GCCTCCCCCAACCAGTGTATGGAAGCCATTGCGACAAACAAGGCTGATGCTGTGACCCTT 234

..******.* *.* ***.* ..*********..*** ..*** ******.********

human GATGGTGGTTTCATATACGAGGCAGGCCTGGCCCCCTACAAACTGCGACCTGTAGCGGCG 297

chimpanzee GATGGCGGTTTCATATACGAGGCAGGCCTGGCCCCCTACAAACTGCGACCTGTAGCGGCG 297

bonobo GATGGCGGTTTCATATACGAGGCAGGCCTGGCCCCCTACAAACTGCGACCTGTAGCGGCG 297

gorilla GATGGCGGTTTCATGTACGAGGCAGGCCTGGACCCCTACAAACTGCGACCTGTAGCGGCG 297

orangutan GATGGCGGTTTGATATACGAGGCAGGCCTGGACCCCTACAAACTGCGACCTGTAGCGGCG 300

CEmacaque GATGGTGGTTTGATGTATGAAGCAGGCCTGGCCCCCTACAAACTGCGACCTGTAGCAGCG 294

Tmacaque GATGGTGGTTTGATGTATGAAGCAGGCCTGGCCCCCTACAAACTGCGACCTGTAGCAGCG 294

baboon GATGGTGGTTTGATGTATGAAGCAGGCCTGGCCCCCCACAAACTGCGACCTGTAGCAGCG 294

mangabey GATGGTGGTTTGATGTATGAAGCAGGCCTGGCCCCCCACAAACTGCGACCTGTAGCAGCG 294

greenM GATGGTGGTTTGATGTACGAAGCAGGCCTGGCCCCCTACAAACTGCGACCTGTAGCAGCG 294

colobus GATGGTGGTTTGATGTACGAAGCAGGACTGGACCCCTACAAACTGCGACCTGTAGCAGCG 294

SNmonkey GATGGTGGTTTGATGTACGAAGCAGGCCTGGACCCCTACAAACTGCGACCTGTAGCAGCG 294

marmoset GATGGCGGTTTGATATATGAGGCAGGCCAGGCCCCCTACCTACTGCGACCGGTAGCAGCA 294

squirrelM GATGGTGGTTTGATATATGAGGCAGGCCAGGCCCCCTACCTACTGCGACCGGTAGCAGCA 294

woollyM GATGGCGGTTTGATATATGAGGCAGGCCAGGCCCCCTACCTACTGCGACCGGTAGCAGCA 294

***** ***** **.** **.*****.*:**.**** **.:********* *****.**.

human GAAGTCTACGGGACCGAAAGACAGCCACGAACTCACTATTATGCCGTGGCTGTGGTGAAG 357

chimpanzee GAAGTCTATGGGACCGAAAGACAGCCACGAACTCACTATTATGCCGTGGCTGTGGTGAAG 357

bonobo GAAGTCTATGGGACCGAAAGACAGCCACGAACTCACTATTATGCCGTGGCTGTGGTGAAG 357

gorilla GAAGTCTACGGGACCGAAAGACAGCCACGAACTCACTATTATGCCGTGGCTGTGGTGAAG 357

orangutan GAAGTCTACGGGACCGAAAGACAGCCACGAACCCACTATTATGCCGTGGCTGTGGTGAAG 360

CEmacaque GAAGTCTACGGGACCGAAGAAAAGCCACGAACCCACTATTATGCCGTGGCTGTGGTGAAG 354

Tmacaque GAAGTCTACGGGACCGAAGAAAAGCCACGAACCCACTATTATGCCGTGGCTGTGGTGAAG 354

baboon GAAGTCTACGGGACCGAAGAAAAGCCACGAACCCACTATTATGCCGTGGCTGTGGTGAAG 354

mangabey GAAGTCTACGGGACTGAAGAAAAGCCACGAACCCACTATTATGCCGTGGCTGTGGTGAAG 354

greenM GAAGTCTACGGGACCGAAGAAAAGCCACGAACCCACTATTATGCCGTGGCTGTGGTGAAG 354

colobus GAAGTCTATGGGACTGAAGGAGAGCCACGAACCCACTATTATGCCGTGGCTGTGGTGAAG 354

SNmonkey GAAGTCTACGGGACTGAAGGAGAGCCACGAACCCACTATTATGCCGTGGCCGTGGTGAAG 354

marmoset GAAGTCTATGGGAGTGAAGCACAGCCACAAACCCACTATTACGCCGTGGCTGTGGTGAAG 354

squirrelM GAAGTCTACGGGAGCGAAGCACAGCCACGAACCCACTATTACGCTGTGGCTGTGGTGAAG 354

woollyM GAAGTCTACGGGAGCAAAGCGCAACCACGAACCCACTATTACGCCGTGGCTGTGGTGAAG 354

******** **** .**. . *.****.*** ******** ** ***** *********

human AAGGGCGGCAGCTTTCAGCTGAACGAACTGCAAGGTCTGAAGTCCTGCCACACAGGCCTT 417

chimpanzee AAGGGCGGCAGCTTTCAGCTGAACGAACTGCAAGGTCTGAAGTCCTGCCACACAGGCCTT 417

bonobo AAGGGCGGCAGCTTTCAGCTGAACGAACTGCAAGGTCTGAAGTCCTGCCACACAGGCCTT 417

gorilla AAGGGCGGCAGCTTTCAGCTGAACGAACTGCAAGGTCTGAAGTCCTGCCACACAGGCCTT 417

orangutan AAGGGCGGCCGCTTTCAGCTGAACGAACTGCAAGGTCTGAAGTCCTGCCACACAGGCCTT 420

CEmacaque AAGGGCGGCCGCTTTCAGCTGAACGAACTGCAAGGTCTGAAGTCCTGCCACACAGGCCTT 414

Tmacaque AAGGGCGGCCGCTTTCAGCTGAACGAACTGCAAGGTCTGAAGTCCTGCCACACAGGCCTT 414

baboon AAGGGCAGCGGCTTTCAGCTGAATGAACTGCAAGGTCTGAAGTCCTGCCACACAGGCCTT 414

mangabey AAGGGCAGCGGCTTTCAGCTGAACGAACTGCAAGGTCTGAAGTCCTGCCACACAGGCCTT 414

greenM AAGGGCGGCCGCTTTCAGCTGAACGAACTGCAAGGTCTGAAGTCCTGCCACACAGGCCTT 414

colobus AAGGGCGGCCGCTTTCAGCTGAACGAACTACAAGGTCTGAAGTCCTGCCACACAGGCCTT 414

SNmonkey AAGGGCGGCCGCTTTCAGCTGAACGAACTACAGGGTCTGAAGTCCTGCCACACAGGCCTT 414

marmoset AAGGGCGGCCGCTTTCAGCTGAACCAGCTGCAAGGTCTGAAGTCCTGCCACACAGGCCTT 414

squirrelM AAGGGCGGCCGCTTTCAGCTGAACCAACTGCAAGGTCTGAAGTCCTGCCACACAGGCCTT 414

woollyM AAGGGTGGCCGCTTTCAGCTGAACCAACTGCAAGGTCTGAAGTCTTGCCACACAGGCCTT 414

***** .** ************* *.**.**.*********** ***************

human CGCAGGACCGCTGGATGGAATGTCCCTATAGGGACACTTCGTCCATTCTTGAATTGGACG 477

chimpanzee CGCAGGACCGCTGGATGGACTGTCCCTATAGGGACACTTCGTCCATTCTTGAATTGGACG 477

bonobo CGCAGGACCGCTGGATGGACTGTCCCTATAGGGACACTTCGTCCATTCTTGAATTGGACG 477

gorilla CGCAGGACCGCTGGATGGAATGTCCCTATAGGGACGCTTCGTCCATTCTTGAATTGGACG 477

orangutan CGCAGGACCGCTGGATGGAATGTCCCTATAGGGACGCTTCGTCCATTCTTGAACTGGACG 480

CEmacaque AACAGGACCGCTGGGTGGATTGTCCCTATAGGGATGCTTCGTCCATTCTTGAACTGGACG 474

Tmacaque AACAGGACCGCTGGGTGGATTGTCCCTATAGGGATGCTTCGTCCATTCTTGAACTGGACG 474

baboon CGCAGGACCGCTGGGTGGAATGTCCCTATAGGGATTCTTCGTCCATTCTTGAACTGGACG 474

mangabey AACAGGACCGCTGGGTGGAATGTCCCTATAGGGATGCTTCGTCCATTCTTGAACTGGATG 474

greenM CGCAGGACCGCTGGGTGGAATGTCCCTATAGGGATGCTTCGTCCATTCTTGAACTGGATG 474

colobus CGCAGGACCGCTGGGTGGAATGTCCCTATAGGGATACTTCGTCCATTCTTGAACTGGATG 474

SNmonkey CGCAGGACTGCTGGGTGGAATGTCCCTATAGGGATGCTTCGTCCATTCTTGAACTGGATG 474

marmoset CGCAGGACTGCTGGGTGGAACATCCCTATAGGGACAATTCGTCCATTCTTGAACTGGACG 474

squirrelM CGCAGGACCGCTGGGTGGAACGTCCCTATAGGGACGATTCGTCCATTCTTAGACTGGTCA 474

woollyM GGCAGGACCGCTGGGTGGAACGTCCCTATAGGGACGATTCGTCCATTCTTGGACTGGATG 474

.****** *****.**** .************ .*************..* ***: .

human GGTCCACCTGAGCCCATTGAGGCAGCTGTGGCCAGGTTCTTCTCAGCCAGCTGTGTTCCC 537

chimpanzee GGTCCACCTGAGCCCATTGAGGCAGCTGTGGCCAGGTTCTTCTCAGCCAGCTGTGTTCCC 537

bonobo GGTCCACCTGAGCCCATTGAGGCAGCTGTGGCCAGGTTCTTCTCAGCCAGCTGTGTTCCC 537

gorilla GGTCCACCTGAGCCCATTGAGGCAGCTGTGGCCAGGTTCTTCTCAGCCAGCTGTGTTCCC 537

orangutan GGTCCACCTGAGCCCATTGAGGCAGCTGTGGCCAGGTTCTTCTCAGCCAGCTGTGTTCCC 540

CEmacaque GGTCCACCTGAGGCCATTGAGGCAGCTGTGGCCAGGTTCTTCTCAGCCAGCTGTGTTCCC 534

Tmacaque GGTCCACCTGAGGCCATTGAGGCAGCTGTGGCCAGGTTCTTCTCAGCCAGCTGTGTTCCC 534

baboon GGTCCACCTGAGCCCATTGAGGCAGCTGTGGCCAGGTTCTTCTCAGCCAGCTGTGTTCCC 534

mangabey GGTCCACCTGAGCCCATTGAGGCAGCTGTGGCCAGGTTCTTCTCAGCCAGCTGTGTTCCC 534

greenM GGTCCACCTGAGCCCATTGAGGCAGCTGTGGCCAGGTTCTTCTCAGCCAGCTGTGTTCCC 534

colobus GGTCCACCTGAGCCCATTGAGGCAGCTGTGGCCAGGTTCTTCTCAGCCAGCTGTGTTCCC 534

SNmonkey GGTCCACCTGAGCCCATTGAGGCAGCTGTGGCCAGGTTCTTCTCAGCCAGCTGTGTTCCC 534

marmoset GGTCCACCTGAGCACATTGAAGCAGCTGTGGCCAGGTTCTTCTCAGCCAGCTGTGTTCCT 534

squirrelM GGTCCACCTGAGCGCATTGAGGCAGCTGTGGCCAGGTTCTTCTCAGCCAGCTGTGTTCCT 534

woollyM GGTCCACCTGAGCGCATTGAGGCAGCTGTGGCCAGGTTCTTCTCAGCCAGCTGTGTTCCT 534

************ ******.**************************************

human GGTGCAGATAAAGGACAGTTCCCCAACCTGTGTCGCCTGTGTGCGGGGACAGGGGAAAAC 597

chimpanzee GGTGCAGATAAAGGACAGTTCCCCAACCTGTGTCGCCTGTGTGCGGGGACAGGGGAAAAC 597

bonobo GGTGCAGATAAAGGACAGTTCCCCAACCTGTGTCGCCTGTGTGCGGGGACAGGGGAAAAC 597

gorilla GGTGCAGATAAAGGACAGTTCCCCAACCTGTGTCGCCTGTGTGCGGGGACAGGGGAAAAC 597

orangutan GGTGCAGATAAAGGACAGTTCCCCAACCTGTGTCGCCTGTGTGCGGGGACAGGGGAAAAC 600

CEmacaque GGTGCAGATAAAGGACAGTTCCCCAACCTGTGTCGCCTGTGTGTGGGGACAGGGGAAAAC 594

Tmacaque GGTGCAGATAAAGGACAGTTCCCCAACCTGTGTCGCCTGTGTGTGGGGACAGGGGAAAAC 594

baboon GGTGCAGATAAAGGACAGTTCCCCAACCTGTGTCGCCTGTGTGTGGGGACAGGGGAAAAC 594

mangabey GGTGCAGATAAAGGACAGTTCCCCAACCTGTGTCGCCTGTGTGCGGGGACAGGGGAAAAC 594

greenM GGTGCAGATGAAGGACAGTTCCCCAACCTGTGTCACCTGTGTGCGGGAACAGGGGAAAAC 594

colobus GGTGCAGATGAAGAACAGTTCCCCAACCTGTGTCGCCTGTGTGCGGGGACAGGGGAAAAC 594

SNmonkey GGTGCAGATAAAGAACAGTTCCCCAACCTGTGTCGCCTGTGTGCGGGGACAGGGGAAAAC 594

marmoset GGTGCAGATGAAAGACAGTTCCCCAACCTATGTCGCCTGTGTGTGGGGAGAGGGGCAAAC 594

squirrelM GGTGCGGATGAAAAACAGTTCCCCAACCTGTGTCGCCTGTGTGTGGGGACAGGAGCAAAC 594

woollyM GGTGTGGATGAAAGACAGTTCCCCAACCTGTGTCGCCTGTGTGTGGGGACAGGGACAAAC 594

**** .***.**..***************.****.******** ***.* ***...****

human AAATGTGCCTTCTCCTCCCAGGAACCGTACTTCAGCTACTCTGGTGCCTTCAAGTGTCTG 657

chimpanzee AACTGTGCCTTCTCCTCCCAGGAACCGTACTTCGGCTACTCTGGTGCCTTCAAGTGTCTG 657

bonobo AACTGTGCCTTCTCCTCCCAGGAACCGTACTTCGGCTACTCTGGTGCCTTCAAGTGTCTG 657

gorilla AAATGTGCCTTCTCCTCCCAGGAACCATACTTCGGCTACTCTGGTGCCTTCAAGTGTCTG 657

orangutan AAATGTGCCTTCTCCTCCCAGGAACCGTACTTCAGTTACTCTGGTGCCTTCAAGTGTCTG 660

CEmacaque AAATGTGCCTTCTCCTCCCAGGAACCGTACTTCGGCTACTCTGGTGCCTTCAAGTGTCTG 654

Tmacaque AAATGTGCCTTCTCCTCCCAGAGACCGTACTTCGGCTACTCTGGTGCCTTCAAGTGTTTG 654

baboon AAATGTGCCTTCTCCTCCCAGGAACCGTACTTTGGCTACTCTGGTGCCTTCAAGTGTCTG 654

mangabey AAATGTGCCTTCTCCTCCCAGGAACCGTACTTCAGCTACTCCGGTGCCTTCAAGTGTCTG 654

greenM AAATGTGCCTTCTCCTCCCAGGAACCGTACTTTGGCTACTCTGGTGCCTTCAAGTGTCTG 654

colobus AAATGTGCCTTCTCCTCCCAGGAACCGTACTTTGGCTACTCTGGTGCCTTCAAGTGTCTG 654

SNmonkey AAATGTGCCTTCTCCTCCCAGGAACCGTACTTCGGCTACTCTGGTGCCTTCAAGTGTCTG 654

marmoset AAATGTGCCTTCTCCTCCAAGGAACCGTACTTCGGCTACTCTGGTGCCTTCAAGTGTCTG 654

squirrelM AAATGTGCCTTCTCCTCCAAGGAACCGTACTTCAGCTACTCTGGGGCCTTCAAGTGTCTG 654

woollyM AAATGTGCCTTTTCCTCCAAGGAACCATACTTTGGCTACTCTGGTGCCTTCAAGTGTCTG 654

**.******** ******.**..***.***** .* ***** ** ************ **

human AGAGACGGGGCTGGAGACGTGGCTTTTATCAGAGAGAGCACAGTGTTTGAGGACCTGTCA 717

chimpanzee AGAGACGGGGCTGGAGACGTGGCTTTTATCAGAGACAGCACAGTGTTTGAGGACCTGTCA 717

bonobo AGAGACGGGGCTGGAGACGTGGCTTTTATCAGAGAGAGCACAGTGTTTGAGGACCTGTCA 717

gorilla AGAGAGGGGGCTGGAGACGTGGCTTTTATCAGAGAGAGCACAGTGTTTGAGGACCTGTCA 717

orangutan AGAGAGGGGGCTGGAGATGTGGCTTTTATCAGAGAGAGCACAGTGTTTGAGGACCTGTCA 720

CEmacaque AGAGACGGCGCTGGAGACGTGGCTTTTATCAGAGAGAGCACAGTGTTTGAGGACCTGTCA 714

Tmacaque AGAGACGGCACTGGAGACGTGGCTTTTATCAGAGAGAGCACAGTGTTTGAGGACCTGTCA 714

baboon AGAGACGGCGCTGGAGACGTGGCTTTTATCAGAGAGAGCACAGTGTTTGAGGACCTGTCA 714

mangabey AGAGACGGCGCTGGAGACGTGGCTTTTATCAGAGAGAGCACAGTGTTTGAGGACCTGTCA 714

greenM AGAGACGGGGCTGGAGACGTGGCTTTTATCAGAGAGAGCACAGTGTTTGAGGACCTGTCA 714

colobus AGGGAGGGGGCTGGAGACGTGGCTTTTATCAGAGAGAGCACAGTGTTTGAGGACCTGTCA 714

SNmonkey AGGGAGGGGGCTGGAGACGTGGCTTTTATCAGAGAGAGCACAGTGTTTGAGGACCTGTCA 714

marmoset AGAGATGGGGCTGGAGACGTGGCTTTTATCAGGGAGAGCACAGTGTTTGAGGACCTGCCA 714

squirrelM AGGGATGGGGCTGGAGACGTGGCTTTTATCAGGGAGAGCACAGTGTTTGAGGACCTGCCA 714

woollyM AGAGACGGGGCTGGAGATGTGGCTTTTATCAGGGAGAGCACAGTGTTTGAGGACCTGCCA 714

**.** ** .******* **************.** ********************* **

human GACGAGGCTGAAAGGGACGAGTATGAGTTACTCTGCCCAGACAACACTCGGAAGCCAGTG 777

chimpanzee GACGAGGCTGAAAGGGACGAGTATGAGTTACTCTGCCCAGACAACACTCGGAAGCCAGTG 777

bonobo GACGAGGCTGAAAGGGACGAGTATGAGTTACTCTGCCCAGACAACACTCGGAAGCCAGTG 777

gorilla GACGAGGCTGAAAGGGATGAGTATGAGTTACTCTGCCCAGACAACACTCGGAAGCCAGTG 777

orangutan GACGAAGCTGAAAGGGACGAGTATGAGCTACTCTGCCCAGACAACACTCGGAAGCCTGTG 780

CEmacaque GACCCCGCTGAAAGGGACAATTATGAGCTGCTCTGCCCAGACAATACTCGGAAGCCAGTG 774

Tmacaque GACCCCGCTGAAAGGGACAATTATGAGCTGCTCTGCCCAGACAATACTCGGAAGCCAGTG 774

baboon GACCCGGCTGAAAGGGACAATTATGAGCTGCTCTGCCCAGACAATACTCGGAAGCCAGTG 774

mangabey GACCCGGCTGAAAGGGACAATTATGAGCTGCTCTGCCCAGACAATACTCGGAAGCCAGTG 774

greenM GACCCGGCTGAAAGGGACAATTATGAGCTGCTCTGCCCAGACAATACTCGGAAGCCAGTG 774

colobus GACCCGGCTGAAAGGGACAATTATGAGCTGCTCTGCCCAGACAATACTCGGAAGCCAGTG 774

SNmonkey GACCCGGCTGAAAGGGATGAGTATGAGCTGCTCTGCCCAGACAATACTCGGAAGCCAGTG 774

marmoset AGCCAGGCTGAAAGGGATGAGTATGAGCTGCTCTGCCCAGACAACACTCGGAAGCCAGTG 774

squirrelM AGCCAGGCTGAAAGGGACGAGTATGAGCTGCTCTGCCCAGACAACACTCGGAAGCCAGTG 774

woollyM AGCCAGGCTGAAAGGGACGAGTATGAGCTGCTCTGCCCAGACAACACTCGGAAGCCAGTG 774

..* . *********** .* ****** *.************** ***********:***

human GACAAGTTCAAAGACTGCCATCTGGCCCGGGTCCCTTCTCATGCCGTTGTGGCACGAAGT 837

chimpanzee GACAAGTTCAAAGACTGCCATCTGGCCCGGGTCCCTTCTCATGCCGTTGTGGCACGAAGT 837

bonobo GACAAGTTCAAAGACTGCCATCTGGCCCGGGTCCCTTCTCATGCCGTTGTGGCACGAAGT 837

gorilla GACAGGTTCAAAGACTGCCATCTGGCCCGGGTCCCTTCTCATGCCGTTGTAGCACGAAGT 837

orangutan GGCAAGTTCAAAGATTGCCATCTGGCCCGGGTCCCTTCTCATGCTGTTGTGGCACGAAGT 840

CEmacaque GACAAGTTCAAGGAGTGCCACCTGGCGCGGGTCCCTTCTCATGCCGTTGTGGCACGAAGT 834

Tmacaque GACAAGTTCAAGGAGTGCCACCTGGCGCGGGTCCCTTCTCATGCCGTTGTGGCACGAAGT 834

baboon GACAAGTTCAAGGAGTGCCACCTGGCGCGGGTCCCTTCTCATGCCGCTGTGGCACGAAGT 834

mangabey GACAAGTTCAAGGAGTGCCACCTGGCGCGGGTCCCTTCTCATGCCGTTGTGGCACGAAGT 834

greenM GACAAGTTCAAGGAGTGCCACCTGGCGCGGGTCCCTTCTCATGCCGTTGTGGCACGAAGT 834

colobus GACAAGTTCAAGGAGTGCCACCTGGCACGGGTCCCTTCTCATGCCGTTGTGGCACGAAGT 834

SNmonkey GACAAGTTCAAGGAGTGCCACCTGGCGCGGGTCCCTTCTCATGCCGTTGTGGCACGAAGT 834

marmoset GATAAGTTTCAGGAATGTCACCTGGCTCGGGTCCCTTCTCATGCCGTTGTGGCACGAAGT 834

squirrelM GACAAGTTCGAGGAGTGCCACCTGGCCCGAGTCCCTTCTCATGCCGTTGTGGCACGAAGT 834

woollyM GACAAGTTCGAGGAGTGCCACCTGGCCCGAGTCCCTTCTCATGCCGTTGTGGCACGAAGT 834

*. *.*** *.** ** ** ***** **.************** * ***.*********

human GTGAATGGCAAGGAGGATGCCATCTGGAATCTTCTCCGCCAGGCACAGGAAAAGTTTGGA 897

chimpanzee GTGAATGGCAAGGAGGATGCCATCTGGAATCTTCTCCGCCAGGCACAGGAAAAGTTTGGA 897

bonobo GTGAATGGCAAGGAGGATGCCATCTGGAATCTTCTCCGCCAGGCACAGGAAAAGTTTGGA 897

gorilla GTGAATGGCAAGGAGGATGCCATCTGGAATCTTCTCCGCCGGGCACAGGAAAAGTTTGGA 897

orangutan GTGAATGGCAAGGAGGACGCCATCTGGGCGCTTCTCCGCAAGGCACAGGAAAAGTTTGGA 900

CEmacaque GTGAACGGCAAGGAGGACGCCATCTGGGAGCTTCTCCGCCAGGCACAGGAGAAGTTTGGA 894

Tmacaque GTGAACGGCAAGGAGGACGCCATCTGGGAGCTTCTCCGCCAGGCACAGGAGAAGTTTGGA 894

baboon GTGAATGGCAAGGAGGACGCCATCTGGGAGCTTCTCCGCCAGTCACAGGAGAAGTTTGGA 894

mangabey GTGAATGGCAAGGAGGACGCCATCTGGGAGCTTCTCCGCCAGTCACAGGAGAAGTTTGGA 894

greenM GTGAACGGCAAGGAGGACGCCATCTGGGAGCTTCTCCGCGAGGCACAGGAGAAGTTTGGA 894

colobus GTGAATGGCATGGAGGACGCCATCTGGGAGCTTCTCCGCCAGGCACAGGAGAAGTTTGGA 894

SNmonkey GTGAATGGCAAGGAGGACGCCATCTGGGAGCTTCTCCGCCAGGCACAGGAGAAGTTTGGA 894

marmoset GGGAACGGCAAGGAGGATGCCATCTGGGAGCTCCTCCGTCGGTCTCAGGAAAAGTTTGGA 894

squirrelM GTGAATGGCAAGGAGGATGCCATCTGGGAGCTCCTCCGCCAGTCTCAGGAAAAGTTTGGA 894

woollyM ATGAACGGCAAGGAGGATGCCATCTGGGAGCTCCTCCGCCAGTCTCAGGAAAAGTTTGGA 894

. *** ****:****** *********.. ** ***** .* *:*****.*********

human AAGGACAAGTCACCGAAATTCCAGCTCTTTGGCTCCCCTAGTGGGCAGAAAGATCTGCTG 957

chimpanzee AAGGACAAGTCACCAAAATTCCAGCTCTTTGGCTCCCCTAGTGGGCAGAAAGATCTGCTG 957

bonobo AAGGACAAGTCACCAAAATTCCAGCTCTTTGGCTCCCCTAGTGGGCAGAAAGATCTGCTG 957

gorilla AAGGACAAGTCACCGAAATTCCAGCTCTTTGGCTCCCCTAGTGGGCAGAAAGATCTGCTG 957

orangutan AAGGACAAGTCAACGAAATTCCAGCTCTTTGGCTCCCCTAGTGGGCAGAAAGATCTGCTG 960

CEmacaque AAGGACAAGTCACCAGAGTTCCAGCTCTTTGGCTCCCCGAGAGGGCAGAAGGATCTGCTG 954

Tmacaque AAGGACAAGTCACCAGAGTTCCAGCTCTTTGGCTCCCCGAGAGGGCAGAAGGATCTGCTG 954

baboon AAGGACAAGTCACCAGCGTTCCAGCTCTTTGGCTCCCCGAGAGGGCAGAAGGATCTGCTG 954

mangabey AAGGACAAGTCACCAGCGTTCCAGCTCTTTGGCTCCCCGAGAGGGCAGAAGGATCTGCTG 954

greenM AAGGACAAGTCACCAGCGTTCCAGCTCTTTGGCTCCCCGAGAGGGCAGAAGGATCTGCTG 954

colobus AAGGACAAGTCACCAGTGTTCCAGCTCTTTGGTTCCCCAAGAGGGCAGAAGGATCTGCTG 954

SNmonkey AAGGACAAGTCACCAGTGTTCCAGCTCTTTGGATCCCCGAGAGGGCAGAAGGATCTGCTG 954

marmoset AAGGACAAGTCACCGGAGTTCCGGCTCTTTGGCTCCCCTAGAGGGGAGAAGGATCTGCTG 954

squirrelM AAGGACAAGTCACCGGCATTCCGGCTCTTTGGCTCCCCTAGCGGGGAGAAGGATCTGCTG 954

woollyM AAGGACGAATCACCAGAGTTCCAGCTCTTTGGCTCCCCTAGCGGGGAGAAGGATCTGCTG 954

******.*.***.*.. .****.********* ***** ** *** ****.*********

human TTCAAGGACTCTGCCATTGGGTTTTCGAGGGTGCCCCCGAGGATAGATTCTGGGCTGTAC 1017

chimpanzee TTCAAGGACTCTGCCATTGGGTTTTCGAGGGTGCCCCCGAGGATAGATTCTGGGCTGTAC 1017

bonobo TTCAAGGACTCTGCCATTGGGTTTTCGAGAGTGCCCCCGAGGATAGATTCTGGGCTGTAC 1017

gorilla TTCAAGGACTCTGCCATTGGGTTTTTGAGGGTGCCCCTGAGGATAGATTCTGGGCTGTAC 1017

orangutan TTCAAGGACTCTGCCATCGGGTTTTCGAGGGTCCCCCCGAGGATAGATTCTGGGCTGTAC 1020

CEmacaque TTCAAGGACTCCGCCATCGGGTTTTCGAGGGTCCCCCTGAGGATCGATTCTGGGCTGTAC 1014

Tmacaque TTCAAGGACTCCGCCATCGGGTTTTCGAGGGTCCCCCTGAGGATCGATTCTGGGCTGTAC 1014

baboon TTCAAGGACTCTGCCATCGGGTTTTCGAGGGTCCCCCCGAGGATCGATTCTGGGCTGTAC 1014

mangabey TTCAAGGACTCCGCCATCGGGTTTTCGAGGGTCCCCCCGAGGATCGATTCTGGGCTGTAC 1014

greenM TTCAAGGACTCCGCCATCGGGTTTTCGAGGGTCCCCCCGAGGATCGATTCTGGGCTGTAC 1014

colobus TTCAAGGACTCCGCCATCGGGTTTTCAAGGGTCCCCCCGAGGATAGATTCTGGGCTGTAC 1014

SNmonkey TTCAAGGACTCTGCCATCGGGTTTTCAAGGGTCCCCCCAAGGATAGATTCTGGGCTGTAC 1014

marmoset TTCAAGGACTCCGCCATCGGGTTTTCCAGGGTCCCTGGGAGGATAGATTCTGGGCTGTAC 1014

squirrelM TTCAAGGACTCCGCCGTCGGGTTTTCCAGGGTCCCTGCGAGGATAGATTCTGGGCTGTAC 1014

woollyM TTCAAGGACTCCGCCATCGGGTTTTCCAGGGTCCCTGCGAGGATAGATTCTGGGCTGTAC 1014

*********** ***.* ******* **.** ** .*****.***************

human CTTGGCTCCGGCTACTTCACTGCCATCCAGAACTTGAGGAAAAGTGAGGAGGAAGTGGCT 1077

chimpanzee CTTGGCTCCAGCTACTTCACTGCCATCCAGAACTTGAGGAAAAGTGAGGAGGAAGTGGCT 1077

bonobo CTTGGCTCCAGCTACTTCACTGCCATCCAGAACTTGAGGAAAAGTGAGGAGGAAGTGGCT 1077

gorilla CTTGGCTCCGGCTACTTCACTGCCATCCAGAACTTGAGGAAAAGTGAGGAGGAAGTGGCT 1077

orangutan CTTGGCTCCGGCTACTTCACTGCCATCCAGAACTTGAGGAATAGTGAGGAGGAAGTGGCT 1080

CEmacaque CTTGGCTCTGGCTACTTGACTGCCATCCAGAACTTGAGGAAAAGTGAGGAGGAGGTGGCT 1074

Tmacaque CTTGGCTCTGGCCACTTGACTGCCATCCAGAACTTGAGGAAAAGTGAGGAGGAGGTGGCT 1074

baboon CTTGGCTCTGGCTACTTGACTGCCATCCAGAACTTGAAGAAAAGTGAGGAGGAGGTGGCT 1074

mangabey CTTGGCTCTGGCTACTTGACTGCCATCCAGAACTTGAAGAAAAGTGAGGAGGAGGTGGCT 1074

greenM CTTGGCTCCGGCTACTTGACTGCCATCCAGAACTTGAAGGAAAGTGAGGAGGAGGTGGCT 1074

colobus CTTGGCTCCGGCTACTTGACTGCCATCCAGAACTTGAGGGAAAGTGAGGAGGAGGTGGCT 1074

SNmonkey CTTGGCTCCGGCTACTTGACTGCCATCCAGAACTTGAGGGAAAGTGAGGAGGAGGTGACT 1074

marmoset CTTGGCTCCAGCTACTTCACCGCCATCCAGAACTTGAGGAAAAGCAAGGAGGAGGTGGCC 1074

squirrelM CTTGGCTCCGGCTACTTCACCGCCATCCAGAACTTGAAGAAAAGCAAGGAGGAGGTGGCT 1074

woollyM CTTGGCTCCGGCTACTTCACTGCCATCCAGAACTTGAGGAAAAGCAAGGAGGAGGTGGCC 1074

******** .** **** ** ****************.*.*:** .*******.***.*

human GCCCGGCGTGCGCGGGTCGTGTGGTGTGCGGTGGGCGAGCAGGAGCTGCGCAAGTGTAAC 1137

chimpanzee GCCCGGCGTGCGCGGGTCGTGTGGTGTGCGGTGGGCGAGCAGGAGCTGCGCAAGTGTAAC 1137

bonobo GCCCGGCGTGCGCGGGTCGTGTGGTGTGCGGTGGGCGAGCAGGAGCTGCGCAAGTGTAAC 1137

gorilla GCCCGGCGTGCGCGGGTCGTGTGGTGTGCGGTGGGCGAGCAGGAGCTGCGCAAGTGTAAC 1137

orangutan GCCCGGCGTGCGCGGGTCGTGTGGTGTGCGGTGGGCGAGCAGGAGCTGCGCAAGTGTAAC 1140

CEmacaque GCCCGGCGTGCGCGAGTCGTGTGGTGTGCAGTGGGCCAGCAGGAGCTGGAAAAGTGTGAC 1134

Tmacaque GCCCGGCGTGCGCGAGTCGTGTGGTGTGCAGTGGGCCAGCAGGAGCTGGAAAAGTGTGAC 1134

baboon GCCCGGCGTGCGCGGGTCGTGTGGTGTGCAGTGGGCCAGCAGGAGCTGGAAAAGTGTGAC 1134

mangabey GCCCGGCGTGCGCGGGTCGTGTGGTGTGCAGTGGGCCAGCAGGAGCTGGAAAAGTGTGAC 1134

greenM GCCCGGCGTGCGCGGGTCGTGTGGTGTGCAGTGGGCCAGCAGGAGCTGGAAAAGTGTGAC 1134

colobus GCCCGGCGTGCGCGGGTCGTGTGGTGTGCAGTGGGTGAGCAGGAGCTGCACAAGTGTAAC 1134

SNmonkey GCCCGGCGTGCGCGGGTCATGTGGTGTGCAGTGGGTGAGGAGGAGCTGCACAAGTGTAAC 1134

marmoset TCCCGGAATGCGCGGGTCGTGTGGTGTGCGGTGGGCGACCAGGAGCAGCGCAAATGTAAC 1134

squirrelM GCCCGGAATGCACGGGTTGTGTGGTGTGCGGTGGGCGACCAGGAGCAGCGCAAATGTAGC 1134

woollyM GCCCGGAATGCGCGGGTCGTGTGGTGTGCGGTGGGCGACCAGGAGCAGCGCAAATGTAGC 1134

*****..***.**.** .**********.***** * ******:* ..**.***..*

human CAGTGGAGTGGCTTGAGCGAAGGCAGCGTGACCTGCTCCTCGGCCTCCACCACAGAGGAC 1197

chimpanzee CAGTGGAGCGGCTTGAGCGAAGGCAGCGTGACCTGCTCCTCGGCCTCCACCACAGAGGAC 1197

bonobo CAGTGGAGCGGCTTGAGCGAAGGCAGCGTGACCTGCTCCTCAGCCTCCACCACAGAGGAC 1197

gorilla CAGTGGAGCAGCTTGAGTGAAGGCAGCGTGACCTGCTCCTCGGCCTCCACCACAGAGGAC 1197

orangutan CAGTGGAGCGGCTTGAGCGAAGGCAGCGTGAACTGCTCCTCGGCCTCCACCACAGAGGAC 1200

CEmacaque CAGTGGAGTGCCCTGAGCGAAGGCAATGTGAACTGCTCCTTGGCCTCCACTGCGGACGAC 1194

Tmacaque CAGTGGAGTGCCCTGAGCGAAGGCAATGTGAACTGCTCCTTGGCCTCCACTGCGGACGAC 1194

baboon CAGTGGAGTGCCCTGAGCGAAGGCAATGTGAACTGCTCCTTGGCCTCCACTGCGGACGAC 1194

mangabey CAGTGGAGTGCCCTGAGCGAAGGCAATGTGAACTGCTCCTTGGCCTCCACTGCGGACGAC 1194

greenM CAGTGGAGTGCCCTGAGTGAAGGCAATGTGAACTGCTCCTTGGCCTCCACTGCGGACGAC 1194

colobus CAGTGGAGTGCCCTGAGCAAAGGCAATGTGAACTGCTCCTCGGCCTCCACTACGGATGAC 1194

SNmonkey CAGTGGAGTGCCCTGAGCAAAGGCAATGTGAACTGCTCCTCGGCCTCCACTACGGATGAC 1194

marmoset CAGTGGAGCAGCCTGAGCGAAGGCAGTGTGACCTGCTCCTCAGCTTCCACCACGGAGGAC 1194

squirrelM CACTGGAGCAGCCTGAGCGAAGGCAGCGTGACCTGCTCCTCGGCCTCTACCGCGGAGGAC 1194

woollyM CAGTGGAGCAGCCTGAGTGAAGGCAGTGTGACCTGCTCCTTGGCCTCCACCGCAGAGGAC 1194

** ***** . * **** .******. ****.******** .** ** ** .*.** ***

human TGCATCGCCCTGGTGCTGAAAGGAGAAGCTGATGCCATGAGTTTGGATGGAGGATATGTG 1257

chimpanzee TGCATCGCCCTGGTGCTGAAAGGAGAAGCTGATGCCATGAGTTTGGATGGAGGATATGTG 1257

bonobo TGCATCGCCCTGGTGCTGAAAGGAGAAGCTGATGCCATGAGTTTGGATGGAGGATATGTG 1257

gorilla TGCATCGCCCTGGTGCTGAAAGGAGAAGCTGATGCCATGAGTTTGGATGGAGGATATGTG 1257

orangutan TGTATCGCCCTGGTGCTGAAAGGAGAAGCTGATGCCATGAGTTTGGATGGAGGATATGTG 1260

CEmacaque TGCATCGCCCTGGTGCTGAAAGGAGAAGCTGATGCCATGAGTTTGGATGGAGGATATGTG 1254

Tmacaque TGCATCGCCCTGGTGCTGAAAGGAGAAGCTGATGCCATGAGTTTGGATGGAGGATATGTG 1254

baboon TGCATCGCCCTGGTGCTGAAAGGAGAAGCTGATGCCATGAGTTTGGATGGAGGATATGTG 1254

mangabey TGCATCGCCCTGGTGCTGAAAGGAGAAGCTGATGCCATGAGTTTGGATGGAGGATATGTG 1254

greenM TGCATCGCCCTGGTGCTGAAAGGAGAAGCTGATGCCATGAGTTTGGATGGAGGATATGTG 1254

colobus TGCATCGCCCTGGTGCTGAAAGGAGAAGCTGATGCCATGAGTTTGGATGGAGGATATGTG 1254

SNmonkey TGCATCGCCCTGGTGCTGAAAGGAGAAGCTGATGCCATGAGTTTGGACGGAGGATATGTG 1254

marmoset TGCATCGCCCTGGTGCTGAAAGGAGAAGCCGATGCCATGAGTTTGGATGGAGGATTTGTC 1254

squirrelM TGCATCGCCCTGGTGCTGAAAGGAGAAGCTGATGCCATGAGTTTGGATGGAGGATTTGTC 1254

woollyM TGCATTGCCTTGGTGCTGAAAGGAGAAGCTGATGCCATGAGTTTGGATGGAGGATTTGTC 1254

** ** *** ******************* ***************** *******:***

human TACACTGCAGGCAAATGTGGTTTGGTGCCTGTCCTGGCAGAGAACTACAAATCCCAACAA 1317

chimpanzee TACACTGCAGGCAAATGTGGTTTGGTGCCTGTCCTGGCAGAGAACTACAAATCCCAACAA 1317

bonobo TACACTGCAGGCAAATGTGGTTTGGTGCCTGTCCTGGCAGAGAACTACAAATCCCAACAA 1317

gorilla TACACTGCAGGCAAATGTGGTTTGGTGCCTGTCCTGGCAGAGAACTACAAATCCCAACAA 1317

orangutan TACACTGCAGGCAAATGTGGTTTGGTGCCTGTCCTGGCAGAGAACTACAAATCCCAACAA 1320

CEmacaque TACACTGCAGGCAAATGTGGTTTGGTGCCTGTCCTGGCAGAGAACTACAAACCCCAACAA 1314

Tmacaque TACACTGCAGGCAAATGTGGTTTGGTGCCTGTCCTGGCAGAGAACTACGAACCCCAACAA 1314

baboon TACACTGCAGGCAAATGTGGTTTGGTGCCTGTCCTGGCAGAGAACTACAAACCCCAACAA 1314

mangabey TACACTGCAGGCAAATGTGGTTTGGTGCCTGTCCTGGCAGAGAACTACAAACCCCAACAA 1314

greenM TACACTGCAGGCAAATGTGGTTTGGTGCCTGTCCTGGCAGAGAACTACAAACCCCAACAA 1314

colobus TACACTGCAGGCAAATGTGGTTTGGTGCCTGTCCTGGCAGAGAACTACAAACCCCAACAA 1314

SNmonkey TACACTGCAGGCAAATGTGGTTTGGTGCCTGTCCTGGCAGAGAACTACAACCCCCAACAA 1314

marmoset TACACTGCAGGCAAATGTGGTTTGGTGCCTGTCCTGGCAGAGAACTACAAATCCCCAAAA 1314

squirrelM TACACTGCAGGCAAATGTGGTTTGGTGCCTGTCCTGGCGGAGAACTACAAATCTCCAAAA 1314

woollyM TACACTGCAGGCAAATGTGGTTTGGTGCCTGTCCTAGCGGAGAACTACAAATCCCCAAAA 1314

***********************************.**.*********.*. * *.*.**

human AGCAGTGACCCTGATCCTAACTGTGTGGATAGACCTGTGGAAGGATATCTTGCTGTGGCG 1377

chimpanzee AGCAGTGACCCTGATCCTAACTGTGTGGATAGACCTGTGGAAGGATATCTTGCTGTGGCG 1377

bonobo AGCAGTGACCCTGATCCTAACTGTGTGGATAGACCTGTGGAAGGATATCTTGCTGTGGCG 1377

gorilla AGCAGTGACCCTGATCCTAACTGTGTGGATAGACCTGTGGAAGGATATCTTGCTGTGGCG 1377

orangutan AGCAGTGACCCTGATCCTAATTGTGTGGATAGACCTGTGGAAGGATATCTTGCTGTGGCA 1380

CEmacaque AGCAGTGGCCCTGATCCTAACTGTGTGGATAGGCCTGTGGAAGGATATCTTGCCGTGGCG 1374

Tmacaque AGCAGTGGCCCTGATCCTAACTGTGTGGATAGGCCTGTGGAAGGATATCTTGCCGTGGCG 1374

baboon AGCAGTGACCCTGATCCTAACTGTGTGGATAGACCTGTGGAAGGATATCTTGCCGTGGCG 1374

mangabey AGCAGTGACCCTGATCCTAACTGTGTGGATAGACCTGTGGAAGGATATCTTGCCGTGGCG 1374

greenM AGCAGTGACCCTGATCCTAACTGTGTGGATAGACCTGTGGAAGGATATCTTGCTGTGGCG 1374

colobus AGCAGTGACCCTGATCCTAACTGTGTGGATAGACCTGTGGAAGGATATCTTGCTGTGGCG 1374

SNmonkey AGCAGTGACCCTGATCCTAACTGTGTGGATAGACCTGTGGAAGGATATCTTGCTGTGGCG 1374

marmoset AGCAGTAACCCTGACCCTAAATGTGTGGATAGACCTGTGGAAGGATATCTTGCTGTGGCG 1374

squirrelM AGCAGTGAACCTGATCCTAATTGTGTGGATAGACCTGTGGAAGGATACCTTGCTGTGGCG 1374

woollyM AGCAGTGACCCCGATGCTAATTGTGTGGATGGACCTGTGGAAGGATATCTCGCTGTGGCG 1374

******...** ** **** *********.*.************** ** ** *****.

human GTGGTTAGGAGATCAGACACTAGCCTTACCTGGAACTCTGTGAAAGGCAAGAAGTCCTGC 1437

chimpanzee GTGGTTAGGAGATCAGACGCTAGCCTTACCTGGAACTCTGTGAAAGGCAAGAAGTCCTGC 1437

bonobo GTGGTTAGGAGATCAGACGCTAGCCTTACCTGGAACTCTGTGAAAGGCAAGAAGTCCTGC 1437

gorilla GTGGTTAGGAGATCAGACACTAGCCTTACCTGGAACTCTGTGAAAGGCAAGAAGTCCTGC 1437

orangutan GTGGTTAGGAAATCAGACGCTAGCCTTACCTGGAACTCTGTGAAAGGCAAGAAGTCCTGC 1440

CEmacaque GTGGTTAGGAATTCAGACGCTGGCCTTACGTGGAACTCTCTGAAAGGCAAGAAGTCCTGC 1434

Tmacaque GTGGTTAGGAATTCAGACGCTGGCCTTACGTGGAACTCTCTGAAAGGCAAGAAGTCCTGC 1434

baboon GTGGTTAGGAATTCAGACGCTGGCCTTACGTGGAACTCTCTGAAAGGCAAGAAGTCCTGC 1434

mangabey GTGGTTAGGAATTCAGACGCTGGCCTTACGTGGAACTCTCTGAAAGGCAAGAAGTCCTGC 1434

greenM GTGGTTAGGAATTCAGACACTGGCCTTACGTGGAACTCTCTGAAAGGCAAGAAGTCCTGC 1434

colobus GTGGTTAGGAGTTCAGACCCTGGCCTTACGTGGAACTCTCTGAAAGGCAAGAAGTCCTGC 1434

SNmonkey GTGGTTAGGAGTTCAGACACTGGCCTTACGTGGAACTCTCTGAAAGGCAAGAAGTCCTGC 1434

marmoset GTGGTTAGGAAATCTGATGCTGGACTTACCTGGAACTCTCTGAAAGGCACAAAGTCCTGC 1434

squirrelM GTGGTTAGGAAATCTGATGCTGGACTTACCTGGAACTCTCTGAAAGGCACAAAGTCCTGC 1434

woollyM GTGGTTAGGAAATCTGACGCTGGACTTACCTGGAACTCTCTGGAAGGCACGAAGTCCTGC 1434

**********.:**:** **.*.***** ********* **.******..*********

human CACACCGCCGTGGACAGGACTGCAGGCTGGAATATCCCCATGGGCCTGCTCTTCAACCAG 1497

chimpanzee CACACCGCCGTGGACAGGACTGCAGGCTGGAATATCCCCATGGGCCTGCTCTTCAACCAG 1497

bonobo CACACCGCCGTGGACAGGACTGCAGGCTGGAATATCCCCATGGGCCTGCTCTTCAACCAG 1497

gorilla CACACCGCCGTGGACAGGACTGCAGGCTGGAATATCCCCATGGGCCTGCTCTTCAACCAG 1497

orangutan CATACCGCCGTGGACAGGACTGCAGGCTGGAATATCCCCATGGGCCTGCTCTTCAACCAG 1500

CEmacaque CACACCGCTGTGGACAGGACTGCAGGCTGGAACATCCCCATAGGCCTGCTCTTCAACCAG 1494

Tmacaque CACACCGCTGTGGACAGGACTGCAGGCTGGAACATCCCCATAGGCCTGCTCTTCAACCAG 1494

baboon CACACCGCCGTGGACAGGACTGCAGGCTGGAACATCCCCATAGGCCTGCTCTTCAACCAG 1494

mangabey CACACCGCCGTGGACAGGACTGCAGGCTGGAACATCCCCATAGGCCTGCTCTTCAACCAG 1494

greenM CACACTGCGGTGGACAGGACTGCAGGCTGGAACATCCCCATAGGCCTGCTCTTCAACCAG 1494

colobus CACACCGCCGTGGACAGGACTGCAGGCTGGAACATCCCCATAGGTCTGCTCTTCAACCAG 1494

SNmonkey CACACAGCCGTGGACAGGACTGCAGGCTGGAACATCCCCATAGGCCTGCTCTTCAACCAG 1494

marmoset CACACCGCGGTGGACAGGACTGCAGGCTGGAACATTCCCATAGGCTTGCTCTTCAACCAG 1494

squirrelM CACACCGCCGTGGACAGGACTGCGGGCTGGAACATTCCCATAGGCTTGCTCTTCAACCAG 1494

woollyM CACACCGCCGTGGGCAGGACTGCAGGCTGGAACATTCCCATAGGCTTGCTCTTCAACCAG 1494

** ** ** ****.*********.******** ** *****.** **************

human ACGGGCTCCTGCAAATTTGATGAATATTTCAGTCAAAGCTGTGCCCCTGGGTCTGACCCG 1557

chimpanzee ACGGGCTCCTGCAAATTTGATGAATATTTCAGTCAAAGCTGTGCCCCTGGGTCTGACCCG 1557

bonobo ACGGGCTCCTGCAAATTTGATGAATATTTCAGTCAAAGCTGTGCCCCTGGGTCTGACCCG 1557

gorilla ACGGGCTCCTGCAAATTTGATGAATATTTCAGTCAAAGCTGTGCCCCTGGGTCTGACCCG 1557

orangutan ACGGGCTCCTGCAAATTTGATGAATACTTCAGTCAAAGCTGTGCCCCTGGGTCTGACCCG 1560

CEmacaque ACGGGCTCCTGCAAATTTGATGAATACTTCAGTCAAAGCTGTGCCCCTGGGGCTGACCCG 1554

Tmacaque ACGGGCTCCTGCAAATTTGATGAATACTTCAGTCAAAGCTGTGCCCCTGGGGCTGACCCG 1554

baboon ACGGGCTCCTGCAAATTTGATGAATACTTCAGTCAAAGCTGTGCCCCTGGGGCTGACCCG 1554

mangabey ACGGGCTCCTGCAAATTTGATGAATACTTCAGTCAAAGCTGTGCCCCTGGGGCTGACCCG 1554

greenM ACGGGCTCCTGCAAATTTGATGAGTACTTCAGTCAAAGCTGTGCCCCTGGGGCTGACCCA 1554

colobus ACGGGCTCCTGCAAATTTGATGAATACTTCAGTCAAAGCTGTGCCCCTGGGGCTGACCCG 1554

SNmonkey ACGGGCTCCTGCAAATTTGATGAATACTTCAGTCAAAGCTGTGCCCCTGGGGCTGACCCG 1554

marmoset ACGGGCTCCTGCAAATTTGATGAATACTTCAGTCAAAGCTGTGCCCCTGGGTCTGACCCG 1554

squirrelM ACGGGCTCCTGCAAATTTGATGAATACTTCAGTCAAAGCTGTGCCCCTGGGGCTGACCCG 1554

woollyM ACGGGCTCCTGCAAATTTGATGAATACTTCAGTCAAAGCTGTGCCCCTGGGTCCGACCCG 1554

***********************.** ************************ * *****.

human AGATCTAATCTCTGTGCTCTGTGTATTGGCGACGAGCAGGGTGAGAATAAGTGCGTGCCC 1617

chimpanzee AGATCTAATCTCTGTGCTCTGTGTATTGGCGACGAGCAGGGTGAGAATAAGTGCGTGCCC 1617

bonobo AGATCTAATCTCTGTGCTCTGTGTATTGGCGACGAGCAGGGTGAGAATAAGTGCGTGCCC 1617

gorilla AGATCTAATCTCTGTGCTCTGTGTATTGGCGACGAGCAGGGTGAGAATAAGTGCGTGCCC 1617

orangutan AGATCTAATCTCTGTGCTCTGTGTATTGGCAACGAGCAGGGTGAGGATAAGTGCGTGCCC 1620

CEmacaque AGATCTAATCTCTGTGCTCTGTGTATTGGCAATGAGCAGGGTGAGGATAAGTGCGTGCCC 1614

Tmacaque AGATCTAATCTCTGTGCTCTGTGTATTGGTAATGAGCAGGGTGAGGATAAGTGCGTGCCC 1614

baboon AGATCTAATCTCTGTGCTCTGTGTATTGGCAATGAGCAGGGTGAGAATAAGTGCGTGCCC 1614

mangabey AGATCTAATCTCTGTGCTCTGTGTATTGGCAATGAGCAGGGTGAGAATAAGTGCGTGCCC 1614

greenM AGATCTAATCTCTGTGCTCTGTGTATTGGCAATGAGCAGGGTGAGAATAAGTGCGTGCCC 1614

colobus AGATCTAATCTCTGTGCTCTGTGTATTGGCAATGAGCAGGGTGAGAATAAGTGCGTGCCC 1614

SNmonkey AGATCTAATCTCTGTGCTCTGTGTATTGGCAATGAGCAGGGTGAGAATAAGTGCGTGCCC 1614

marmoset AAATCTAATCTCTGTGCTCTGTGTATTGGCAACGAGCAGGGTGAGAATAAGTGCGTGCCC 1614

squirrelM AAATCTAATCTCTGTGCTCTGTGTGTTGGCAATGAGCAGGGTGAGAATAAGTGTGCGCCC 1614

woollyM AAATCTAATCTCTGTGCTCTGTGTATTGGCAACGAGCAGGGTGAGAATAAGTGCGTGCCC 1614

*.**********************.**** .* ************.******* * ****

human AACAGCAACGAGAGATACTACGGCTACACTGGGGCTTTCCGGTGCCTGGCTGAGAATGCT 1677

chimpanzee AACAGCAATGAGAGATACTACGGCTACACTGGGGCTTTCCGGTGCCTGGCTGAGAATGCT 1677

bonobo AACAGCAATGAGAGATACTACGGCTACACTGGGGCTTTCCGGTGCCTGGCTGAGAATGCT 1677

gorilla AACAGCAATGAGAGATACTACGGCTACACTGGGGCTTTCCGGTGCCTGGCTGAGAATGCT 1677

orangutan AACAGCAATGAGAGATACTACGGCTACACTGGGGCTTTCCGGTGCCTGGCTGAGAATGCT 1680

CEmacaque AACAACAATGAGAGATACTACGGCTACACTGGGGCTTTCCGGTGCCTGGCTGAGAATGCT 1674

Tmacaque AACACCAATGAGAGATACTACGGCTACACTGGGGCTTTCCGGTGCCTAGCTGAGAATGCT 1674

baboon AACAAAAGTGAGAGATACTACGGCTACACTGGGGCTTTCCGGTGCCTGGCTGAGAATGCT 1674

mangabey AACAAAAGTGAGAGATACTACGGCTACACTGGGGCTTTCCGGTGCCTGGCTGAGAATGCT 1674

greenM AACAACAATGAGAGATACTACGGCTACAATGGGGCTTTCCGGTGCCTGGCTGAGAATGCT 1674

colobus AACAGCAATGAGAGATACTACGGCTACAATGGGGCTTTCCGGTGCCTGGCCGAGAATGCT 1674

SNmonkey AACAGCAATGAGAGATACTACGGCTACAATGGGGCTTTCCGGTGCCTGGCCGAGAATGCT 1674

marmoset AAGAGCAATGAGAGATACTATGGCTACGATGGGGCTTTCCGGTGCCTGGCTGAGAATGCT 1674

squirrelM AACAGCAATGAGAGATACTATGGCTACGACGGGGCTTTCCGGTGCCTGGCTGAGAATGCT 1674

woollyM AACAGCAATGAGAGATACTACGGCTACGATGGGGCTTTCCGGTGCCTGGCTGAGAATGCT 1674

** * .*. *********** ******.. *****************.** *********

human GGAGACGTTGCATTTGTGAAAGATGTCACTGTCTTGCAGAACACTGATGGAAATAACAAT 1737

chimpanzee GGAGACGTTGCATTTGTGAAAGATGTCACTGTCTTGCAGAACACTGATGGAAATAACAGT 1737

bonobo GGAGACGTTGCATTTGTGAAAGATGTCACTGTCTTGCAGAACACTGATGGAAATAACAGT 1737

gorilla GGAGACGTTGCATTTGTGAAAGATGTCACTGTCTTGCAGAACACTGATGGAAATAACAAT 1737

orangutan GGAGACGTTGCATTTGTGAAAGATGTCACAGTCTTGCAGAACACTGATGGAAATAACACT 1740

CEmacaque GGAGACGTTGCGTTTGTGAAAGATGTCACTGTCTTGCAGAACACTGATGGAAAGAACACT 1734

Tmacaque GGAGACGTTGCGTTTGTGAAAGATGTCACTGTCTTGCAGAACACTGATGGAAAGAACACT 1734

baboon GGAGACGTTGCGTTTGTGAAAGATGTCACTGTCTTGCAGAACACTGATGGAAAGAACACT 1734

mangabey GGAGACGTTGCGTTTGTGAAAGATGTCACTGTCTTGCAGAACACTGATGGAAAGAACACT 1734

greenM GGAGACGTTGCGTTTGTGAAAGATGTCACCGTCTTGCAGAACACTGATGGAAAGAACACT 1734

colobus GGAGATGTTGCGTTTGTAAAAGATGTCACTGTCTTGCAGAACACTGATGGAAAGAACACT 1734

SNmonkey GGAGACGTTGCATTTGTGAAAGATGTCACTGTCTTGCAGAACACTGAGGGAAAGAACACT 1734

marmoset GGGGACGTTGCCTTCGTGAAAGATGCCACTGTCTTGCAGAACACCGACGGAAAGAACACT 1734

squirrelM GGGGATGTTGCCTTCGTGAAAGATACCACTGTCTTGCAGAACACCAATGGAAAGAACACT 1734

woollyM GGGGATGTTGCATTCGTGAAAGAGGCCACCGTCTTGCAGAACACCAATGGAAAGAACACT 1734

**.** ***** ** **.***** . *** ************** .* ***** **** *

human GAGGCATGGGCTAAGGATTTGAAGCTGGCAGACTTTGCGCTGCTGTGCCTCGATGGCAAA 1797

chimpanzee GAGGCATGGGCTAAGGATTTGAAGCTGGCAGACTTTGCGCTGCTGTGCCTCGATGGCAAA 1797

bonobo GAGGCATGGGCTAAGGATTTGAAGCTGGCGGACTTTGCGCTGCTGTGCCTCGATGGCAAA 1797

gorilla GAGGCATGGGCTAAGGATTTGAAGCTGGCGGACTTTGTGCTGCTGTGCCTCGATGGCAAA 1797

orangutan GAGCCATGGGCTAAGGATTTGAAGCTGGAGGACTTTGAGCTGCTGTGCCTCGATGGCAAA 1800

CEmacaque GACGCATGGGCTAAGGATTTGAAGCTGAACGACTTTGAGCTGCTGTGCCTCGATGGCACG 1794

Tmacaque GACGCATGGGCTAAGGATTTGAAGCTGAACGACTTTGAGCTGCTGTGCCTCGATGGCACG 1794

baboon GAAGCATGGGCTAAGGATTTGAAGCTGAACGACTTTGAGCTGCTGTGCCTCGATGGCACG 1794

mangabey GAAGCATGGGCTAAGGATTTGAAGCTGAACGACTTTGAGCTGCTGTGCCTCGATGGCACG 1794

greenM GAAGCATGGGCGAAGGATTTGAAGCTGAACGACTTTGAGCTGCTGTGCCTCGATGGCACG 1794

colobus GAAGCATGGGCTAAGGATTTGAAGCTGCAGGACTTTGAGCTGCTGTGCCTTGATGGCACG 1794

SNmonkey GAAGCATGGGCTAAGGATTTGAAGCTGCAGGACTTTGCGCTGCTGTGCCTTGATGGCACG 1794

marmoset GAAGCATGGGCTAAGGATTTGAAGCTGGAGAACTTTGAGCTGCTGTGCCTTGATGGCACC 1794

squirrelM GAAGCGTGGGCTAAGGATTTGAAGCTGGAGAACTTTGAGCTGCTGTGCCTCGATGGTACC 1794

woollyM GATGCATGGGCTAAGGATTTGAAGCTGGAGGACTTTGAGCTGCTGTGCCTCGATGGCACC 1794

** *.***** *************** . .****** ************ ***** *.

human CGGAAGCCTGTGACTGAGGCTAGAAGCTGCCATCTTGCCATGGCCCCGAATCATGCCGTG 1857

chimpanzee CGGAAGCCTGTGACTGAGGCTAGAAGCTGCCATCTTGCCATGGCCCCGAATCATGCCGTG 1857

bonobo CGGAAGCCTGTGACTGAGGCTAGAAGCTGCCATCTTGCCATGGCCCCGAATCATGCCGTG 1857

gorilla CAGAAGCCTGTGACTGAGGCTAGAAGTTGCCATCTTGCCATGGCCCCGAATCATGCCGTG 1857

orangutan CGGAAGCGTGTGACTGAGGCTAGAAGCTGCCATCTCGCCATGGCCCCAAATCATGCCGTG 1860

CEmacaque CGGAAGCCTGTGACTGAGGCTAGGAGCTGCCATCTCGCCATGGCCCCGAATCATGCCGTG 1854

Tmacaque CGGAAGCCTGTGACTGAGGCTAGGAGCTGCCATCTCGCCATGGCCCCGAATCATGCCGTG 1854

baboon CGGAAGCCTGTGACTGAGGCTAGGAGCTGCCATCTCGCCACGGCCCCGAATCATGCCGTG 1854

mangabey CGGAAGCCTGTGATTGAGGCTAGGAGCTGCCATCTCGCCACGGCCCCGAATCATGCCGTG 1854

greenM CGGAAGCCTGTGACTGAGGCTAGGAGCTGCCATCTCGCCATGGCCCCGAATCATGCCGTG 1854

colobus CGGAAGCCTGTGACTGAGGCTAGGAGCTGCCATCTCGCCATGGCCCCGAATCATGCCGTG 1854

SNmonkey CGGAAGCCTGTGACTGAGGCTAGGAGCTGCCATCTCGCCATGGCCCCGAATCATGCCGTG 1854

marmoset CGGAAGCCTGTTACAGAGGCTAGGAGCTGCCATCTTGCCATGGCCCCGAATCATGCCGTG 1854

squirrelM CGGAAGCCTGTGACGGAGGCTAAGAGCTGCCATCTTGCCATGGCCCCAAATCATGCCGTG 1854

woollyM CGGAAACCTGTGACGGAGGCTAAGAGCTGCCATCTTGCCATGGCTCCGAATCATGCCGTG 1854

*.***.* *** * *******..** ******** **** *** **.************

human GTGTCTCGGATGGATAAGGTGGAACGCCTGAAACAGGTGTTGCTCCACCAACAGGCTAAA 1917

chimpanzee GTGTCTCGGACGGATAAGGTGGAACGCCTGAAACAGGTGCTGCTCCACCAACAGGCTAAA 1917

bonobo GTGTCTCGGACGGATAAGGTGGAACGCCTGAAACAGGTGCTGCTCCACCAACAGGCTAAA 1917

gorilla GTGTCTCGGACGGATAAGGTGGAACGCCTGAAACAGGTGCTGCTCCACCAACAGGCTAAA 1917

orangutan GTGTCTCGGATAGATAAGGTGGAACGCCTGACACAGGTGCTGCTCCACCAACAGGCTAAA 1920

CEmacaque GTGTCTCGGACAGATAAGGTGGAACGCCTGAAACAGGTGCTGTTCGACCAACAGGCTAAA 1914

Tmacaque GTGTCTCGGACAGAAAAGGTGGAACGCCTGAAACAGGTGCTGTTCGACCAACAGGCTAAA 1914

baboon GTGTCTCGGACAGATAAGGTGGAACGCCTGAAACAGGTGCTGTTCGACCAACAGGCTAAA 1914

mangabey GTGTCTCGGACAGATAAGGTGGAACGCCTGAAACAGGTGCTGTTCGACCAACAGGCTAAA 1914

greenM GTGTCTCGGACAGATAAGGTGGAACGCCTGAAACAGGTGCTGTTCGACCAACAGGCTAAA 1914

colobus GTGTCTCGGACGGATAAGGTGGAACGCCTGAAACAGGTGCTGTTCGACCAACAGGCTAAA 1914

SNmonkey GTGTCTCGGACGGATAAGGTGGAACGCCTGAAACAGGTGCTGTTCGACCAACAGGCTAAA 1914

marmoset GTGTCTCGGATGGATAAGGTGGAACGCCTCAAACAGGTGCTGTTCCAGCAGCAGGCTAAA 1914

squirrelM GTGTCTCGGACGGATAAGGTGGAACGCCTGAAACAGGTGCTGTTCCAGCAACAGGCTAAA 1914

woollyM GTGTCTCGGGTGGATAAGGTGGAACGCCTGAAACAGGTGCTGTTCCAGCAACAGGCTAAA 1914

*********. .**:************** *.******* ** ** * **.*********

human TTTGGGAGAAATGGATCTGACTGCCCGGACAAGTTTTGCTTATTCCAGTCTGAAACCAAA 1977

chimpanzee TTTGGGAGAAATGGATCTGACTGCCCGGAGAAGTTTTGCTTATTCCGGTCTGAAACCAAA 1977

bonobo TTTGGGAGAAATGGATCTGACTGCCCGGACAAGTTTTGCTTATTCCGGTCTGAAACCAAA 1977

gorilla TTTGGGAGAAATGGATCTGACTGCCCGGACAAGTTTTGCTTATTCCGGTCTGAAACCAAA 1977

orangutan TTTGGGAGAAATGGATCTGATTGCCCGGACAAGTTTTGCTTATTCCGGTCTGAAACCAAA 1980

CEmacaque TTTGGGAAAAATGGATCTGACTGCCCGGGCACATTTTGCTTATTCCAGTCTAAAACCAAA 1974

Tmacaque TTTGGGAAAAATGGATCTGACTGCCCGGGCACATTTTGCTTATTCCAGTCTAAAACCAAA 1974

baboon TTTGGGAAAAATGGATCTGACTGCCCAGGCACATTTTGCTTATTCCAGTCGAAAACCAAA 1974

mangabey TTTGGGAAAAATGGATCTGACTGCCCGGGCACATTTTGCTTATTCCAGTCGAAAACCAAA 1974

greenM TTTGGGAAAAATGGATCTGGCTGCCCGGGCACGTTTTGCTTATTCCAGTCTAAAACCAAA 1974

colobus TTTGGGAGAAATGGATCTGACTGCCCGGGCACGTTTTGCTTATTCCAGTCTAAAACCAAA 1974

SNmonkey TTTGGGAGAAATGGATCTGACTGCCCGGGCACGTTTTGCTTATTCCAGTCTAAAACCAAA 1974

marmoset TTTGGGAGAAATGGATCTGCCTGCCCGGGCAAGTTTTGCTTATTCCATTCCGAAACCAAA 1974

squirrelM TTTGGAACAAATGGATCTGACTGCCCAGACAAGTTTTGCTTATTCCGGTCTGAAACCAAA 1974

woollyM TTTGGGAGAAATGGATCTGACTGCCCGGGCAAGTTTTGCTTATTCCAGTCTGAAACCAAA 1974

*****.* *********** *****.*. *..*************. ** .********

human AACCTTCTGTTCAATGACAACACTGAGTGTCTGGCCAGACTCCATGGCAAAACAACATAT 2037

chimpanzee AACCTTCTGTTCAATGACAACACTGAGTGTCTGGCCAGACTCCATGGCAAAACAACATAT 2037

bonobo AACCTTCTGTTCAATGACAACACTGAGTGTCTGGCCAGACTCCATGGCAAAACAACATAT 2037

gorilla AACCTTCTGTTCAATGACAACACTGAGTGTCTGGCCAGACTCCATGGCAAAACAACATAT 2037

orangutan AACCTTCTGTTCAATGACAACACTGAGTGTCTGGCCAGACTCCATGGCAAAACAACATAT 2040

CEmacaque AACCTTCTGTTCAATGACAACACTGAGTGTCTGGCCAGACTCCATGGCAAAACAACATAT 2034

Tmacaque AACCTTCTGTTCAATGACAACACTGAGTGTCTGGCCAGACTCCATGGCAAAACAACATAT 2034

baboon AACCTTCTGTTCAATGACAACACTGAGTGTCTGGCCAGACTCCATGGCAAAACAACATAT 2034

mangabey AACCTTCTGTTCAATGACAACACTGAGTGTCTGGCCAGACTCCATGGCAAAACAACATAT 2034

greenM AACCTTCTGTTCAATGACAACACTGAGTGCCTGGCCAGACTCCATGGCAAAACAACATAT 2034

colobus AACCTTCTGTTTAATGACAACACTGAGTGTCTGGCCAGACTCCATGGCAAAACAACGTAT 2034

SNmonkey AACCTTCTGTTCAGTGACAACACTGAGTGTCTGGCCAGACTCCATGGCAAAACAACGTAT 2034

marmoset AACCTTCTGTTCAATGACAACACTGAGTGTCTGGCCAGACTCCACGGCAAAACAACATAT 2034

squirrelM AACCTTCTATTCAATGATAACACTGAGTGTCTGGCCGGACTCCAGGGCAAAACAACATAT 2034

woollyM AACCTTCTGTTCAATGACAACACTGAGTGTCTGGCTGGACTCCATGGCAAAACAACATAT 2034

********.** *.*** *********** ***** .******* ***********.***

human GAAAAATATTTGGGACCACAGTATGTCGCAGGCATTACTAATCTGAAAAAGTGCTCAACC 2097

chimpanzee GAAAAATATTTGGGACCACAGTATGTCGCAGCCATTACTAATCTGAAAAAGTGCTCAACC 2097

bonobo GAAAAATATTTGGGACCACAGTATGTCGCAGCCATTACTAATCTGAAAAAGTGCTCAACC 2097

gorilla GAAAAATATTTGGGACCACAGTATGTCACAGCCATTACTAATCTGAAAAAGTGCTCAACC 2097

orangutan GAAAAATATTTGGGACCACAGTATGTCGCAGCTATTACTAATCTGAAAAAGTGCTCAACC 2100

CEmacaque GAAAAATATTTGGGACCACAGTATGTCACAGCCATTACTAATCTGAAAAAGTGCTCAAGC 2094

Tmacaque GAAAAATATTTGGGACCACAGTATGTCACAGCCATTACTAATCTGAAAAAGTGCTCAAGC 2094

baboon GAAAAATATTTGGGACCACAGTATGTCACAGCCATTACTAATCTGAAAAAGTGCTCAAGC 2094

mangabey GAAAAATATTTGGGACCACAGTATGTCACAGCCATTACTAATCTGAAAAAGTGCTCAAGC 2094

greenM GAAAAATATTTGGGACCACAGTATGTCACAGCCGTTACTAATCTGAAAAAGTGCTCAACC 2094

colobus GAAAAATATTTGGGACCGCAGTATGTCACAGCCATTACTAATCTGAAAAAGTGCTCAACC 2094

SNmonkey GAAAAATATTTGGGACCACAGTATGTCACAGCCATTGCTAATCTGAAAAAGTGCTCAACC 2094

marmoset GAAAAATATTTGGGACCCCAGTATGTCACAGCCATTAATAATCTGAAAAAGTGCTCAACC 2094

squirrelM GAAAAATATTTGGGACCACAGTATGTCACAGCCATTAATAATCTGAAAAAGTGCTCAACC 2094

woollyM GAAAAATATTTGGGACCACAGTATGTCACAGCCATTAATAATCTGAAAAAGTGCTCAACC 2094

***************** *********.*** .**..******************** *

human TCCCCCCTCCTGGAAGCCTGTGAATTCCTCAGGAAGTAA 2136

chimpanzee TCCCCCCTCCTGGAAGCCTGTGAATTCCTCAGGAAGTAA 2136

bonobo TCCCCCCTCCTGGAAGCCTGTGAATTCCTCAGGAAGTAA 2136

gorilla TCCCCCCTCCTGGAAGCCTGTGAATTCCTCAGGAAGTAA 2136

orangutan TCCCCCCTCCTGGAAGCCTGTGCATTCCTCAGGAAGTAA 2139

CEmacaque TCCCCCCTCCTGGAAGCCTGTGCATTCCTCCAGAAGTAA 2133

Tmacaque TCCCCCCTCCTGGAAGCCTGTGCATTCCTCCAGAAGTAA 2133

baboon TCCCCCCTCCTGGAAGCCTGTGCGTTCCTCCAGAAGTAA 2133

mangabey TCCCCCCTCCTGGAAGCCTGTGCGTTCCTCCAGAAGTAA 2133

greenM TCCCCCCTCCTGGAAGCCTGTGCATTCCTCCAGAAGTAA 2133

colobus TCCCCCCTCCTGGAAGCTTGTGCATTCCTCAGGAAGTAA 2133

SNmonkey TCCCCCCTCCTGGAAGCCTGTGCATTCCTCAGGAAGTAA 2133

marmoset TCCCCTCTCCTGGAGGCCTGTGCGTTCCTTAGGAAGTAA 2133

squirrelM TCCCCCCTCCTGGAAGCCTGCGCGTTCCTTAGGAAGTAA 2133

woollyM TCCCCCCTCCTGGAAGCCTGCGCGTTCCTCAGGAAGTAA 2133

***** ********.** ** *..***** ..*******

Lactoferrin protein: CLUSTAL O(1.2.1) multiple sequence alignment.

**Positions displaying evidence of positive selection are denoted in green.*

human MKLVFLVLLFLGALGLCLAGRRR-RSVQWCAVSQPEATKCFQWQRNMRKVRGPPVSCIKR 59

chimpanzee MKLVFLVLLFLGALGLCLAGPRR-RSVQWCTVSQPEATKCFRWQRNMRRVRGPPVSCIKR 59

bonobo MKLVFLVLLFLGALGLCLAGPRR-RSVQWCTVSQPEATKCFRWQRNMRRVRGPPVSCIKR 59

gorilla MKLVFPVLLFLGALGLCLAAPRR-RSVRWCTVSQPEATKCFRWQRNMKRVRGPPVSCIKR 59

orangutan MKLVFSALLFLGALGLCLAAPRRRRSVRWCTVSQPEATKCFRWQRNMRRVRGPPVSCIKT 60

CEmacaque MKLVFLALLFLGTLGLCLAA--RRRSVRWCAVSKPEATKCSQWQRNLRRVRGPPVSCIKR 58

Tmacaque MKLVFLALLFLGTLGLCLAA--RRRSVRWCAVSKPEATKCSQWQRNLRRVRGPPVSCIKR 58

baboon MKLVFLALLFLGTLGLCLAA--RRRSVRWCAVSKPEATKCSQWQRNLRRVRGPPVSCIKR 58

mangabey MKLVFLALLFLGTLGLCLAA--RRRSVRWCAVSKPEATKCSQWQRNLRSVRGPPVSCIKR 58

AGM MKLVFLALLFLGTLGLCLAA--RRRSVRWCTVSQPEATKCSQWQGNLRRVRGPPVSCIKR 58

colobus MKLAFLALLFLGTLGLCLAA--RRRSVRWCTVSQPEATKCSQWQRNLRRVLGPPVSCIKR 58

SNmonkey MKLVFLALLFLGTLGLCLAA--RRRSVRWCTVSQPEATKCSQWQRNLRRVRGPPVSCIKR 58

marmoset MKLVFPTLLFLGALGLCLAAPR--GSVRWCTVSKPEATKCIQWQRNLRKVGGPSVSCIKR 58

squirrelM MKLVFPALLFLGALGLCLAAPR--GSVRWCTVSKPEATKCIQWQRNLRKVGGPPVSCIKR 58

woollyM MKLVFPALLFLGALGLCLAAPR--GSVRWCTVSKPEATKCIKWQRNLRNVGGPFVSCIKR 58

***.* .*****:******. **:**:**:****** :** *:: * ** *****

human DSPIQCIQAIAENRADAVTLDGGFIYEAGLAPYKLRPVAAEVYGTERQPRTHYYAVAVVK 119

chimpanzee DSPIQCIQAIAENRADAVTLDGGFIYEAGLAPYKLRPVAAEVYGTERQPRTHYYAVAVVK 119

bonobo DSPIQCIQAIAENRADAVTLDGGFIYEAGLAPYKLRPVAAEVYGTERQPRTHYYAVAVVK 119

gorilla DSPIQCIQAIAENRADAVTLDGGFMYEAGLDPYKLRPVAAEVYGTERQPRTHYYAVAVVK 119

orangutan DSPTQCIQAIAANRADAVTLDGGLIYEAGLDPYKLRPVAAEVYGTERQPRTHYYAVAVVK 120

CEmacaque ASPTNCIQAIAANRADAMTLDGGLMYEAGLAPYKLRPVAAEVYGTEEKPRTHYYAVAVVK 118

Tmacaque ASPTNCIQAIAANRADAMTLDGGLMYEAGLAPYKLRPVAAEVYGTEEKPRTHYYAVAVVK 118

baboon ASPTNCIRAIAANRADAMTLDGGLMYEAGLAPHKLRPVAAEVYGTEEKPRTHYYAVAVVK 118

mangabey ASPTNCIRAIAANRADAMTLDGGLMYEAGLAPHKLRPVAAEVYGTEEKPRTHYYAVAVVK 118

AGM ASPTNCIQAIAANKADAMTLDGGLMYEAGLAPYKLRPVAAEVYGTEEKPRTHYYAVAVVK 118

colobus ASPTNCIQAIATNKADAVTLDGGLMYEAGLDPYKLRPVAAEVYGTEGEPRTHYYAVAVVK 118

SNmonkey ASPTKCIQAIATNEADAVTLDGGLMYEAGLDPYKLRPVAAEVYGTEGEPRTHYYAVAVVK 118

marmoset ASPTQCVEAIATNKADAVTLDGGLIYEAGQAPYLLRPVAAEVYGSEAQPQTHYYAVAVVK 118

squirrelM TSPTQCMEAIATNKADAVTLDGGLIYEAGQAPYLLRPVAAEVYGSEAQPRTHYYAVAVVK 118

woollyM ASPNQCMEAIATNKADAVTLDGGLIYEAGQAPYLLRPVAAEVYGSKAQPRTHYYAVAVVK 118

** :*:.*** *.***:*****::**** *: **********:: :*:**********

human KGGSFQLNELQGLKSCHTGLRRTAGWNVPIGTLRPFLNWTGPPEPIEAAVARFFSASCVP 179

chimpanzee KGGSFQLNELQGLKSCHTGLRRTAGWTVPIGTLRPFLNWTGPPEPIEAAVARFFSASCVP 179

bonobo KGGSFQLNELQGLKSCHTGLRRTAGWTVPIGTLRPFLNWTGPPEPIEAAVARFFSASCVP 179

gorilla KGGSFQLNELQGLKSCHTGLRRTAGWNVPIGTLRPFLNWTGPPEPIEAAVARFFSASCVP 179

orangutan KGGRFQLNELQGLKSCHTGLRRTAGWNVPIGTLRPFLNWTGPPEPIEAAVARFFSASCVP 180

CEmacaque KGGRFQLNELQGLKSCHTGLNRTAGWIVPIGMLRPFLNWTGPPEAIEAAVARFFSASCVP 178

Tmacaque KGGRFQLNELQGLKSCHTGLNRTAGWIVPIGMLRPFLNWTGPPEAIEAAVARFFSASCVP 178

baboon KGSGFQLNELQGLKSCHTGLRRTAGWNVPIGILRPFLNWTGPPEPIEAAVARFFSASCVP 178

mangabey KGSGFQLNELQGLKSCHTGLNRTAGWNVPIGMLRPFLNWMGPPEPIEAAVARFFSASCVP 178

AGM KGGRFQLNELQGLKSCHTGLRRTAGWNVPIGMLRPFLNWMGPPEPIEAAVARFFSASCVP 178

colobus KGGRFQLNELQGLKSCHTGLRRTAGWNVPIGILRPFLNWMGPPEPIEAAVARFFSASCVP 178

SNmonkey KGGRFQLNELQGLKSCHTGLRRTAGWNVPIGMLRPFLNWMGPPEPIEAAVARFFSASCVP 178

marmoset KGGRFQLNQLQGLKSCHTGLRRTAGWNIPIGTIRPFLNWTGPPEHIEAAVARFFSASCVP 178

squirrelM KGGRFQLNQLQGLKSCHTGLRRTAGWNVPIGTIRPFLDWSGPPERIEAAVARFFSASCVP 178

woollyM KGGRFQLNQLQGLKSCHTGLGRTAGWNVPIGTIRPFLDWMGPPERIEAAVARFFSASCVP 178

**. ****:*********** ***** :*** :****:* **** ***************

human GADKGQFPNLCRLCAGTGENKCAFSSQEPYFSYSGAFKCLRDGAGDVAFIRESTVFEDLS 239

chimpanzee GADKGQFPNLCRLCAGTGENNCAFSSQEPYFGYSGAFKCLRDGAGDVAFIRDSTVFEDLS 239

bonobo GADKGQFPNLCRLCAGTGENNCAFSSQEPYFGYSGAFKCLRDGAGDVAFIRESTVFEDLS 239

gorilla GADKGQFPNLCRLCAGTGENKCAFSSQEPYFGYSGAFKCLREGAGDVAFIRESTVFEDLS 239

orangutan GADKGQFPNLCRLCAGTGENKCAFSSQEPYFSYSGAFKCLREGAGDVAFIRESTVFEDLS 240

CEmacaque GADKGQFPNLCRLCVGTGENKCAFSSQEPYFGYSGAFKCLRDGAGDVAFIRESTVFEDLS 238

Tmacaque GADKGQFPNLCRLCVGTGENKCAFSSQRPYFGYSGAFKCLRDGTGDVAFIRESTVFEDLS 238

baboon GADKGQFPNLCRLCVGTGENKCAFSSQEPYFGYSGAFKCLRDGAGDVAFIRESTVFEDLS 238

mangabey GADKGQFPNLCRLCAGTGENKCAFSSQEPYFSYSGAFKCLRDGAGDVAFIRESTVFEDLS 238

AGM GADEGQFPNLCHLCAGTGENKCAFSSQEPYFGYSGAFKCLRDGAGDVAFIRESTVFEDLS 238

colobus GADEEQFPNLCRLCAGTGENKCAFSSQEPYFGYSGAFKCLREGAGDVAFIRESTVFEDLS 238

SNmonkey GADKEQFPNLCRLCAGTGENKCAFSSQEPYFGYSGAFKCLREGAGDVAFIRESTVFEDLS 238

marmoset GADERQFPNLCRLCVGRGANKCAFSSKEPYFGYSGAFKCLRDGAGDVAFIRESTVFEDLP 238

squirrelM GADEKQFPNLCRLCVGTGANKCAFSSKEPYFSYSGAFKCLRDGAGDVAFIRESTVFEDLP 238

woollyM GVDERQFPNLCRLCVGTGTNKCAFSSKEPYFGYSGAFKCLRDGAGDVAFIRESTVFEDLP 238

*.*: ******:**.* * *:*****:.***.*********:*:*******:*******

human DEAERDEYELLCPDNTRKPVDKFKDCHLARVPSHAVVARSVNGKEDAIWNLLRQAQEKFG 299

chimpanzee DEAERDEYELLCPDNTRKPVDKFKDCHLARVPSHAVVARSVNGKEDAIWNLLRQAQEKFG 299

bonobo DEAERDEYELLCPDNTRKPVDKFKDCHLARVPSHAVVARSVNGKEDAIWNLLRQAQEKFG 299

gorilla DEAERDEYELLCPDNTRKPVDRFKDCHLARVPSHAVVARSVNGKEDAIWNLLRRAQEKFG 299

orangutan DEAERDEYELLCPDNTRKPVGKFKDCHLARVPSHAVVARSVNGKEDAIWALLRKAQEKFG 300

CEmacaque DPAERDNYELLCPDNTRKPVDKFKECHLARVPSHAVVARSVNGKEDAIWELLRQAQEKFG 298

Tmacaque DPAERDNYELLCPDNTRKPVDKFKECHLARVPSHAVVARSVNGKEDAIWELLRQAQEKFG 298

baboon DPAERDNYELLCPDNTRKPVDKFKECHLARVPSHAAVARSVNGKEDAIWELLRQSQEKFG 298

mangabey DPAERDNYELLCPDNTRKPVDKFKECHLARVPSHAVVARSVNGKEDAIWELLRQSQEKFG 298

AGM DPAERDNYELLCPDNTRKPVDKFKECHLARVPSHAVVARSVNGKEDAIWELLREAQEKFG 298

colobus DPAERDNYELLCPDNTRKPVDKFKECHLARVPSHAVVARSVNGMEDAIWELLRQAQEKFG 298

SNmonkey DPAERDEYELLCPDNTRKPVDKFKECHLARVPSHAVVARSVNGKEDAIWELLRQAQEKFG 298

marmoset SQAERDEYELLCPDNTRKPVDKFQECHLARVPSHAVVARSGNGKEDAIWELLRRSQEKFG 298

squirrelM SQAERDEYELLCPDNTRKPVDKFEECHLARVPSHAVVARSVNGKEDAIWELLRQSQEKFG 298

woollyM SQAERDEYELLCPDNTRKPVDKFEECHLARVPSHAVVARSMNGKEDAIWELLRQSQEKFG 298

. ****:************* :*::**********.**** ** ***** ***.:*****

human KDKSPKFQLFGSPSGQKDLLFKDSAIGFSRVPPRIDSGLYLGSGYFTAIQNLRKSEEEVA 359

chimpanzee KDKSPKFQLFGSPSGQKDLLFKDSAIGFSRVPPRIDSGLYLGSSYFTAIQNLRKSEEEVA 359

bonobo KDKSPKFQLFGSPSGQKDLLFKDSAIGFSRVPPRIDSGLYLGSSYFTAIQNLRKSEEEVA 359

gorilla KDKSPKFQLFGSPSGQKDLLFKDSAIGFLRVPLRIDSGLYLGSGYFTAIQNLRKSEEEVA 359

orangutan KDKSTKFQLFGSPSGQKDLLFKDSAIGFSRVPPRIDSGLYLGSGYFTAIQNLRNSEEEVA 360

CEmacaque KDKSPEFQLFGSPRGQKDLLFKDSAIGFSRVPLRIDSGLYLGSGYLTAIQNLRKSEEEVA 358

Tmacaque KDKSPEFQLFGSPRGQKDLLFKDSAIGFSRVPLRIDSGLYLGSGHLTAIQNLRKSEEEVA 358

baboon KDKSPAFQLFGSPRGQKDLLFKDSAIGFSRVPPRIDSGLYLGSGYLTAIQNLKKSEEEVA 358

mangabey KDKSPAFQLFGSPRGQKDLLFKDSAIGFSRVPPRIDSGLYLGSGYLTAIQNLKKSEEEVA 358

AGM KDKSPAFQLFGSPRGQKDLLFKDSAIGFSRVPPRIDSGLYLGSGYLTAIQNLKESEEEVA 358

colobus KDKSPVFQLFGSPRGQKDLLFKDSAIGFSRVPPRIDSGLYLGSGYLTAIQNLRESEEEVA 358

SNmonkey KDKSPVFQLFGSPRGQKDLLFKDSAIGFSRVPPRIDSGLYLGSGYLTAIQNLRESEEEVT 358

marmoset KDKSPEFRLFGSPRGEKDLLFKDSAIGFSRVPGRIDSGLYLGSSYFTAIQNLRKSKEEVA 358

squirrelM KDKSPAFRLFGSPSGEKDLLFKDSAVGFSRVPARIDSGLYLGSGYFTAIQNLKKSKEEVA 358

woollyM KDESPEFQLFGSPSGEKDLLFKDSAIGFSRVPARIDSGLYLGSGYFTAIQNLRKSKEEVA 358

**:* *:***** *:*********:** *** **********.::******::*:***:

human ARRARVVWCAVGEQELRKCNQWSGLSEGSVTCSSASTTEDCIALVLKGEADAMSLDGGYV 419

chimpanzee ARRARVVWCAVGEQELRKCNQWSGLSEGSVTCSSASTTEDCIALVLKGEADAMSLDGGYV 419

bonobo ARRARVVWCAVGEQELRKCNQWSGLSEGSVTCSSASTTEDCIALVLKGEADAMSLDGGYV 419

gorilla ARRARVVWCAVGEQELRKCNQWSSLSEGSVTCSSASTTEDCIALVLKGEADAMSLDGGYV 419

orangutan ARRARVVWCAVGEQELRKCNQWSGLSEGSVNCSSASTTEDCIALVLKGEADAMSLDGGYV 420

CEmacaque ARRARVVWCAVGQQELEKCDQWSALSEGNVNCSLASTADDCIALVLKGEADAMSLDGGYV 418

Tmacaque ARRARVVWCAVGQQELEKCDQWSALSEGNVNCSLASTADDCIALVLKGEADAMSLDGGYV 418

baboon ARRARVVWCAVGQQELEKCDQWSALSEGNVNCSLASTADDCIALVLKGEADAMSLDGGYV 418

mangabey ARRARVVWCAVGQQELEKCDQWSALSEGNVNCSLASTADDCIALVLKGEADAMSLDGGYV 418

AGM ARRARVVWCAVGQQELEKCDQWSALSEGNVNCSLASTADDCIALVLKGEADAMSLDGGYV 418

colobus ARRARVVWCAVGEQELHKCNQWSALSKGNVNCSSASTTDDCIALVLKGEADAMSLDGGYV 418

SNmonkey ARRARVMWCAVGEEELHKCNQWSALSKGNVNCSSASTTDDCIALVLKGEADAMSLDGGYV 418

marmoset SRNARVVWCAVGDQEQRKCNQWSSLSEGSVTCSSASTTEDCIALVLKGEADAMSLDGGFV 418

squirrelM ARNARVVWCAVGDQEQRKCSHWSSLSEGSVTCSSASTAEDCIALVLKGEADAMSLDGGFV 418

woollyM ARNARVVWCAVGDQEQRKCSQWSSLSEGSVTCSLASTAEDCIALVLKGEADAMSLDGGFV 418

:*.***:*****::* .**.:**.**:*.*.** ***::*******************:*

human YTAGKCGLVPVLAENYKSQQSSDPDPNCVDRPVEGYLAVAVVRRSDTSLTWNSVKGKKSC 479

chimpanzee YTAGKCGLVPVLAENYKSQQSSDPDPNCVDRPVEGYLAVAVVRRSDASLTWNSVKGKKSC 479

bonobo YTAGKCGLVPVLAENYKSQQSSDPDPNCVDRPVEGYLAVAVVRRSDASLTWNSVKGKKSC 479

gorilla YTAGKCGLVPVLAENYKSQQSSDPDPNCVDRPVEGYLAVAVVRRSDTSLTWNSVKGKKSC 479

orangutan YTAGKCGLVPVLAENYKSQQSSDPDPNCVDRPVEGYLAVAVVRKSDASLTWNSVKGKKSC 480

CEmacaque YTAGKCGLVPVLAENYKPQQSSGPDPNCVDRPVEGYLAVAVVRNSDAGLTWNSLKGKKSC 478

Tmacaque YTAGKCGLVPVLAENYEPQQSSGPDPNCVDRPVEGYLAVAVVRNSDAGLTWNSLKGKKSC 478

baboon YTAGKCGLVPVLAENYKPQQSSDPDPNCVDRPVEGYLAVAVVRNSDAGLTWNSLKGKKSC 478

mangabey YTAGKCGLVPVLAENYKPQQSSDPDPNCVDRPVEGYLAVAVVRNSDAGLTWNSLKGKKSC 478

AGM YTAGKCGLVPVLAENYKPQQSSDPDPNCVDRPVEGYLAVAVVRNSDTGLTWNSLKGKKSC 478

colobus YTAGKCGLVPVLAENYKPQQSSDPDPNCVDRPVEGYLAVAVVRSSDPGLTWNSLKGKKSC 478

SNmonkey YTAGKCGLVPVLAENYNPQQSSDPDPNCVDRPVEGYLAVAVVRSSDTGLTWNSLKGKKSC 478

marmoset YTAGKCGLVPVLAENYKSPKSSNPDPKCVDRPVEGYLAVAVVRKSDAGLTWNSLKGTKSC 478

squirrelM YTAGKCGLVPVLAENYKSPKSSEPDPNCVDRPVEGYLAVAVVRKSDAGLTWNSLKGTKSC 478

woollyM YTAGKCGLVPVLAENYKSPKSSDPDANCVDGPVEGYLAVAVVRKSDAGLTWNSLEGTKSC 478

****************: :** ** :*** ************ ** .*****::*.***

human HTAVDRTAGWNIPMGLLFNQTGSCKFDEYFSQSCAPGSDPRSNLCALCIGDEQGENKCVP 539

chimpanzee HTAVDRTAGWNIPMGLLFNQTGSCKFDEYFSQSCAPGSDPRSNLCALCIGDEQGENKCVP 539

bonobo HTAVDRTAGWNIPMGLLFNQTGSCKFDEYFSQSCAPGSDPRSNLCALCIGDEQGENKCVP 539

gorilla HTAVDRTAGWNIPMGLLFNQTGSCKFDEYFSQSCAPGSDPRSNLCALCIGDEQGENKCVP 539

orangutan HTAVDRTAGWNIPMGLLFNQTGSCKFDEYFSQSCAPGSDPRSNLCALCIGNEQGEDKCVP 540

CEmacaque HTAVDRTAGWNIPIGLLFNQTGSCKFDEYFSQSCAPGADPRSNLCALCIGNEQGEDKCVP 538

Tmacaque HTAVDRTAGWNIPIGLLFNQTGSCKFDEYFSQSCAPGADPRSNLCALCIGNEQGEDKCVP 538

baboon HTAVDRTAGWNIPIGLLFNQTGSCKFDEYFSQSCAPGADPRSNLCALCIGNEQGENKCVP 538

mangabey HTAVDRTAGWNIPIGLLFNQTGSCKFDEYFSQSCAPGADPRSNLCALCIGNEQGENKCVP 538

AGM HTAVDRTAGWNIPIGLLFNQTGSCKFDEYFSQSCAPGADPRSNLCALCIGNEQGENKCVP 538

colobus HTAVDRTAGWNIPIGLLFNQTGSCKFDEYFSQSCAPGADPRSNLCALCIGNEQGENKCVP 538

SNmonkey HTAVDRTAGWNIPIGLLFNQTGSCKFDEYFSQSCAPGADPRSNLCALCIGNEQGENKCVP 538

marmoset HTAVDRTAGWNIPIGLLFNQTGSCKFDEYFSQSCAPGSDPKSNLCALCIGNEQGENKCVP 538

squirrelM HTAVDRTAGWNIPIGLLFNQTGSCKFDEYFSQSCAPGADPKSNLCALCVGNEQGENKCAP 538

woollyM HTAVGRTAGWNIPIGLLFNQTGSCKFDEYFSQSCAPGSDPKSNLCALCIGNEQGENKCVP 538

**** ********:***********************:**:*******:*:****:**.*

human NSNERYYGYTGAFRCLAENAGDVAFVKDVTVLQNTDGNNNEAWAKDLKLADFALLCLDGK 599

chimpanzee NSNERYYGYTGAFRCLAENAGDVAFVKDVTVLQNTDGNNSEAWAKDLKLADFALLCLDGK 599

bonobo NSNERYYGYTGAFRCLAENAGDVAFVKDVTVLQNTDGNNSEAWAKDLKLADFALLCLDGK 599

gorilla NSNERYYGYTGAFRCLAENAGDVAFVKDVTVLQNTDGNNNEAWAKDLKLADFVLLCLDGK 599

orangutan NSNERYYGYTGAFRCLAENAGDVAFVKDVTVLQNTDGNNTEPWAKDLKLEDFELLCLDGK 600

CEmacaque NNNERYYGYTGAFRCLAENAGDVAFVKDVTVLQNTDGKNTDAWAKDLKLNDFELLCLDGT 598

Tmacaque NTNERYYGYTGAFRCLAENAGDVAFVKDVTVLQNTDGKNTDAWAKDLKLNDFELLCLDGT 598

baboon NKSERYYGYTGAFRCLAENAGDVAFVKDVTVLQNTDGKNTEAWAKDLKLNDFELLCLDGT 598

mangabey NKSERYYGYTGAFRCLAENAGDVAFVKDVTVLQNTDGKNTEAWAKDLKLNDFELLCLDGT 598

AGM NNNERYYGYNGAFRCLAENAGDVAFVKDVTVLQNTDGKNTEAWAKDLKLNDFELLCLDGT 598

colobus NSNERYYGYNGAFRCLAENAGDVAFVKDVTVLQNTDGKNTEAWAKDLKLQDFELLCLDGT 598

SNmonkey NSNERYYGYNGAFRCLAENAGDVAFVKDVTVLQNTEGKNTEAWAKDLKLQDFALLCLDGT 598

marmoset KSNERYYGYDGAFRCLAENAGDVAFVKDATVLQNTDGKNTEAWAKDLKLENFELLCLDGT 598

squirrelM NSNERYYGYDGAFRCLAENAGDVAFVKDTTVLQNTNGKNTEAWAKDLKLENFELLCLDGT 598

woollyM NSNERYYGYDGAFRCLAENAGDVAFVKEATVLQNTNGKNTDAWAKDLKLEDFELLCLDGT 598

:..****** *****************:.******:*:*.: ******* :* ******.

human RKPVTEARSCHLAMAPNHAVVSRMDKVERLKQVLLHQQAKFGRNGSDCPDKFCLFQSETK 659

chimpanzee RKPVTEARSCHLAMAPNHAVVSRTDKVERLKQVLLHQQAKFGRNGSDCPEKFCLFRSETK 659

bonobo RKPVTEARSCHLAMAPNHAVVSRTDKVERLKQVLLHQQAKFGRNGSDCPDKFCLFRSETK 659

gorilla QKPVTEARSCHLAMAPNHAVVSRTDKVERLKQVLLHQQAKFGRNGSDCPDKFCLFRSETK 659

orangutan RKRVTEARSCHLAMAPNHAVVSRIDKVERLTQVLLHQQAKFGRNGSDCPDKFCLFRSETK 660

CEmacaque RKPVTEARSCHLAMAPNHAVVSRTDKVERLKQVLFDQQAKFGKNGSDCPGTFCLFQSKTK 658

Tmacaque RKPVTEARSCHLAMAPNHAVVSRTEKVERLKQVLFDQQAKFGKNGSDCPGTFCLFQSKTK 658

baboon RKPVTEARSCHLATAPNHAVVSRTDKVERLKQVLFDQQAKFGKNGSDCPGTFCLFQSKTK 658

mangabey RKPVIEARSCHLATAPNHAVVSRTDKVERLKQVLFDQQAKFGKNGSDCPGTFCLFQSKTK 658

AGM RKPVTEARSCHLAMAPNHAVVSRTDKVERLKQVLFDQQAKFGKNGSGCPGTFCLFQSKTK 658

colobus RKPVTEARSCHLAMAPNHAVVSRTDKVERLKQVLFDQQAKFGRNGSDCPGTFCLFQSKTK 658

SNmonkey RKPVTEARSCHLAMAPNHAVVSRTDKVERLKQVLFDQQAKFGRNGSDCPGTFCLFQSKTK 658

marmoset RKPVTEARSCHLAMAPNHAVVSRMDKVERLKQVLFQQQAKFGRNGSACPGKFCLFHSETK 658

squirrelM RKPVTEAKSCHLAMAPNHAVVSRTDKVERLKQVLFQQQAKFGTNGSDCPDKFCLFRSETK 658

woollyM RKPVTEAKSCHLAMAPNHAVVSRVDKVERLKQVLFQQQAKFGRNGSDCPGKFCLFQSETK 658

:* * **:***** ********* :*****.***:.****** *** ** .****:*:**

human NLLFNDNTECLARLHGKTTYEKYLGPQYVAGITNLKKCSTSPLLEACEFLRK 711

chimpanzee NLLFNDNTECLARLHGKTTYEKYLGPQYVAAITNLKKCSTSPLLEACEFLRK 711

bonobo NLLFNDNTECLARLHGKTTYEKYLGPQYVAAITNLKKCSTSPLLEACEFLRK 711

gorilla NLLFNDNTECLARLHGKTTYEKYLGPQYVTAITNLKKCSTSPLLEACEFLRK 711

orangutan NLLFNDNTECLARLHGKTTYEKYLGPQYVAAITNLKKCSTSPLLEACAFLRK 712

CEmacaque NLLFNDNTECLARLHGKTTYEKYLGPQYVTAITNLKKCSSSPLLEACAFLQK 710

Tmacaque NLLFNDNTECLARLHGKTTYEKYLGPQYVTAITNLKKCSSSPLLEACAFLQK 710

baboon NLLFNDNTECLARLHGKTTYEKYLGPQYVTAITNLKKCSSSPLLEACAFLQK 710

mangabey NLLFNDNTECLARLHGKTTYEKYLGPQYVTAITNLKKCSSSPLLEACAFLQK 710

AGM NLLFNDNTECLARLHGKTTYEKYLGPQYVTAVTNLKKCSTSPLLEACAFLQK 710

colobus NLLFNDNTECLARLHGKTTYEKYLGPQYVTAITNLKKCSTSPLLEACAFLRK 710

SNmonkey NLLFSDNTECLARLHGKTTYEKYLGPQYVTAIANLKKCSTSPLLEACAFLRK 710

marmoset NLLFNDNTECLARLHGKTTYEKYLGPQYVTAINNLKKCSTSPLLEACAFLRK 710

squirrelM NLLFNDNTECLAGLQGKTTYEKYLGPQYVTAINNLKKCSTSPLLEACAFLRK 710

woollyM NLLFNDNTECLAGLHGKTTYEKYLGPQYVTAINNLKKCSTSPLLEACAFLRK 710

****.******* *:**************:.: ******:******* **:*
